# Detecting pattern transitions in psychological time series – A validation study on the Pattern Transition Detection Algorithm (PTDA)

**DOI:** 10.1371/journal.pone.0265335

**Published:** 2022-03-11

**Authors:** Kathrin Viol, Helmut Schöller, Andreas Kaiser, Clemens Fartacek, Wolfgang Aichhorn, Günter Schiepek

**Affiliations:** 1 Institute of Synergetics and Psychotherapy Research, University Hospital of Psychiatry, Psychotherapy and Psychosomatics, Paracelsus Medical University, Salzburg, Austria; 2 Institute for Clinical Psychology, University Hospital of Psychiatry, Psychotherapy and Psychosomatics, Paracelsus Medical University, Salzburg, Austria; 3 Department of Psychology, Ludwig Maximilian University of Munich, Munich, Germany; Technical University of Madrid, SPAIN

## Abstract

With the increasing use of real-time monitoring procedures in clinical practice, psychological time series become available to researchers and practitioners. An important interest concerns the identification of pattern transitions which are characteristic features of psychotherapeutic change. Change Point Analysis (CPA) is an established method to identify the point where the mean and/or variance of a time series change, but changes of other and more complex features cannot be detected by this method. In this study, an extension of the CPA, the Pattern Transition Detection Algorithm (PTDA), is optimized and validated for psychological time series with complex pattern transitions. The algorithm uses the convergent information of the CPA and other methods like Recurrence Plots, Time Frequency Distributions, and Dynamic Complexity. These second level approaches capture different aspects of the primary time series. The data set for testing the PTDA (300 time series) is created by an instantaneous control parameter shift of a simulation model of psychotherapeutic change during the simulation runs. By comparing the dispersion of random change points with the real change points, the PTDA determines if the transition point is significant. The PTDA reduces the rate of false negative and false positive results of the CPA below 5% and generalizes its application to different types of pattern transitions. RQA quantifiers also can be used for the identification of nonstationary transitions in time series which was illustrated by using Determinism and Entropy. The PTDA can be easily used with Matlab and is freely available at Matlab File Exchange (https://www.mathworks.com/matlabcentral/fileexchange/80380-pattern-transition-detection-algorithm-ptda).

## Introduction

With the increasing use of real-time monitoring devices like the Synergetic Navigation System [1], high frequency sampled longitudinal data of psychological processes become available. Fields of research include, for example, psychotherapy processes [2, 3], developmental psychology [4, 5], unconscious processes [6], treatment outcome [7, 8], psychotraumatology [9], sudden gains and losses in psychotherapy [10–12], treatment of multi-problem families [13], psychological aspects of immune diseases [14] or epilepsy [15, 16], and suicidal crises [17, 18]. Real-time monitoring produces time series with daily or even within-day measurements of psychological variables relevant to psychotherapeutic changes like emotions, motivation, insights, and symptom severity.

The availability of such time series open the possibility to go beyond the assessment of pre-post changes of psychotherapy and to shed light on the mechanisms of change that are still largely unknown [19, 20]. The temporal sequences of psychological variables in high frequency sampled time series allows to derive causality [21] by investigating what has happened in the hours or days before a change occurs. One crucial prerequisite to derive mechanisms of change from empirical time series is to identify the point of change in a patient’s individual process in a reliable and valid way. This is non-trivial due to the noisy, discontinuous, and complex patterns of the time series [22, 23].

Besides investigating mechanisms of change, data from real-time monitoring can be used to identify early warning signals that can, once validated, inform the therapist about the current state of a patient. This information can be used for example to set specific interventions [24, 25], or, in the most extreme case, prevent suicide attempts [18]. In order to identify early warning signals, one has to determine the point of change in the time series first. Those retrospective assessments might lead to more reliable predictors of change than are currently available.

It should be noted that long-term predictions are impossible in complex nonlinear systems, but with regard to short-term predictions, several indicators of qualitative changes of a system have been identified. Therefore, researchers and therapists will not be successful in long-term forecasting of specific trajectories of change, but should be vigilant to the possibility of transition points of the system [26]. Such transitions from one pattern to another are called *phase transitions* [24]. In general, a phase transition has occurred when the system has shifted to a *qualitatively different behavior*, i.e., when a change from one phase to another has occurred, where a *phase* refers to a specific stable pattern. The existence of such tipping points (“criticality”), where transitions to a different state occur, characterize nonlinear dynamic systems irrespective of the system under investigation. Theories of self-organization like Synergetics [24, 27] describe how such phase transitions are governed by underlying control parameters, i.e., variables that change on a slower time scale and influence the functional relation between the observed microscopic or macroscopic variables.

A variety of measures to determine the point at which a system changes its qualitative behavior exist in various disciplines like climate research, physics, ecology, neuroscience, and physiology (e.g., [28–30], see [26] for an overview). A commonly used method in psychology but also in other research areas is the Change Point Analysis [31], which is available in softwares like R and Matlab. The Change Point Analysis (CPA) is able to identify one or more points in a time series where pre-defined statistical properties as the mean or the variance change. One of the aims of this study is to test its applicability to short time series of psychotherapy processes with changing complex patterns.

As stated above, however, a phase transition is defined as a qualitative change of the system. This is not restricted to a change of the mean of a time series, but can concern any pattern. Nonlinear time series are often analyzed by two classical methods, the identification of the (local) Largest Lyapunov Exponent [32–34] or the (pointwise) Correlation Dimension [32, 35, 36]. Unfortunately, these measures are not suitable for the relatively short time series which usually are created by real-time monitoring or ecological momentary assessment of psychotherapeutic processes because they require considerably longer time series (> 1000 sampling points) than are usually available in daily ratings in psychotherapy (~ 100 sampling points). Several alternative indicators of change have been reported in [28] and [30]. Among those are again a multitude of measures that require long time series, but also methods that can be applied to psychological data, such as changes of the frequency, variance, distribution, and autocorrelation.

The application of these entities to detect changes in a time series requires to extend the methods in a way that captures the dynamics. This can be done by calculating these measures for small segments of a time series (sliding window approach). For the frequency, this exists in terms of *Time Frequency Distributions* [37], and in the combined dynamics of fluctuation and distribution as it is captured by the *Dynamic Complexity* measure [24, 38]. Temporal change of autocorrelations can be identified in a more sophisticated way that extends to repeating patterns by *Recurrence Plots*. An important spectrum of advanced and sophisticated methods is available by Recurrence Quantification Analysis (RQA) [39, 40], which allows for the identification of dynamic features (e.g., determinism or entropy) in time series [41], of patterns in multidimensional time series data [42–44], phase synchronization in networks [45], chimera states [46] or coupling in bivariate and multivariate dynamics [47]. The quantifiers can be used to detect pattern transitions in dynamic systems (e.g., [41]). We will apply CPA to Recurrence Plots as a contributor to PTDA and also use known quantifiers (determinism and entropy) for reasons of cross-validation (see Results).

The results of these measures are a new time series (Dynamic Complexity), a matrix with time on the horizontal axis and the frequencies of the vertical axis (Time Frequency Distributions), and again a matrix with time on both axes (Recurrence Plots). We will call these “secondary measures” in the following, in contrast to the original “primary” time series. These secondary time series or matrices capture different aspects of the primary time series that have shown to be relevant for detecting phase transitions. Change Point Analysis can then be applied not only to detect changes of the primary time series, but also to detect changes of the distribution, frequency, and recurrent patterns. Using information from various secondary measures increases the validity of the location of change [26].

The assessments described above have been implemented in an easy-to-use algorithm called *Pattern Transition Detection Algorithm* (PTDA), where the user simply enters a time series. The program will then either inform the user that no change point was found or on the time-related location of the pattern transition.

The aim of this study is (1) to assess the performance of the CPA on time series with changes of complex patterns, (2) to extend the CPA to identify changes of the second order features like distribution, fluctuation, frequency, and autocorrelation, and (3) to validate the new PTDA in terms of precision, rate of false negative results, and rate of false positive results. Furthermore, we will investigate the performance with increasing levels of noise, with the transition points placed at different positions along the time series, and with prolonged (i.e., not sudden) periods of change. Finally, the PTDA will be applied to empirical data.

Compared with the first description of the algorithm [26], we illustrate in the present paper not only the feasibility of applying the PTDA to some simulated and empirical data, but conduct a systematic validation of the algorithm by using 300 simulated time series. Furthermore, we optimized the algorithm. Some methods proved to be not sensitive enough for the identification of transitions and, in consequence, were eliminated, as Instantaneous Frequency and Permutation Entropy. Synchronization Pattern Analysis refers to the identification of changing inter-item correlations during the process and is a promising indicator of critical periods before transitions occur. However, the hypothesis of increased synchronization as a precursor of transitions needs further investigation in empirical system dynamics and for this, it was not included in this step of PTDA development. Here, we included fewer converging methods and applied the Change Point Analysis (CPA) also to the Time Frequency Distributions included in the PTDA. For this, the aim of this study is far beyond the aim of the Schiepek et al. paper.

## Methods

The idea of using different methods to gain a convergent validation for a pattern transition was first presented and explained in detail in [26]. It is based on the CPA introduced by Killick et al. [31]. We will present how these methods are combined in a single algorithm, the PTDA. In detail, we will explain the steps of the algorithm in order to improve the rate of precision, and to reduce false positive and false negative results. The paper will also present the results of the evaluation of the PTDA using simulated time series and its applicability to empirical data.

In the following, we will refer to a *change point* (CP) as a point of the time series that was applied to a single method and is the result of the CPA, and to a *transition point* (TP) as a point that was identified by the *Pattern Transition Detection Algorithm* (PTDA), which incorporates the results of all change points found for the different methods.

### Data for validation

For the validation of the PTDA, 300 time series were simulated with a mathematical model of psychotherapeutic processes. In the sense of an illustrative pilot application, the PTDA was also tested on four empirical time series.

#### The model

The algorithm was developed and validated on simulated time series produced by a theoretical computational model of psychotherapeutic change. The details can be found in [48–51]. In short, the model includes five variables (order parameters), which are connected by nonlinear functions, and four control parameters, which modulate the shape of the functions, i.e., they determine the strength and the shape of the impact of the variables onto each other. The five variables represent common client-related factors of the psychotherapeutic process: problem severity (*P*), emotions (*E*), insight (*I*), therapeutic success (*S*), and motivation for change (*M*), which change on a relatively fast time scale (hours to days). The four parameters, on the other hand, represent relatively stable trait characteristics of a client: alliance with the therapist/ability to bond or attach (*a*), cognitive competencies (*c*), motivation to cope with problems/hopefulness (*m*), and resources/behavioral skills (*r*). The parameters can be seen as dispositions or traits that change on a slower time scale (weeks to months) and are intended to be changed during psychotherapy (personality development). In other words, psychotherapy works in this model by increasing the trait parameters, which influence the dynamics of the state variables [50]. The computational model includes 9 coupled nonlinear difference equations which are shown in the Supplement 2 in S1 File. The numerical values of the resulting time series are created by iterative application of the map onto the respective last discrete value (x_t-1_, with t = discrete time steps).

#### Simulating pattern transitions

**Fig 1:**
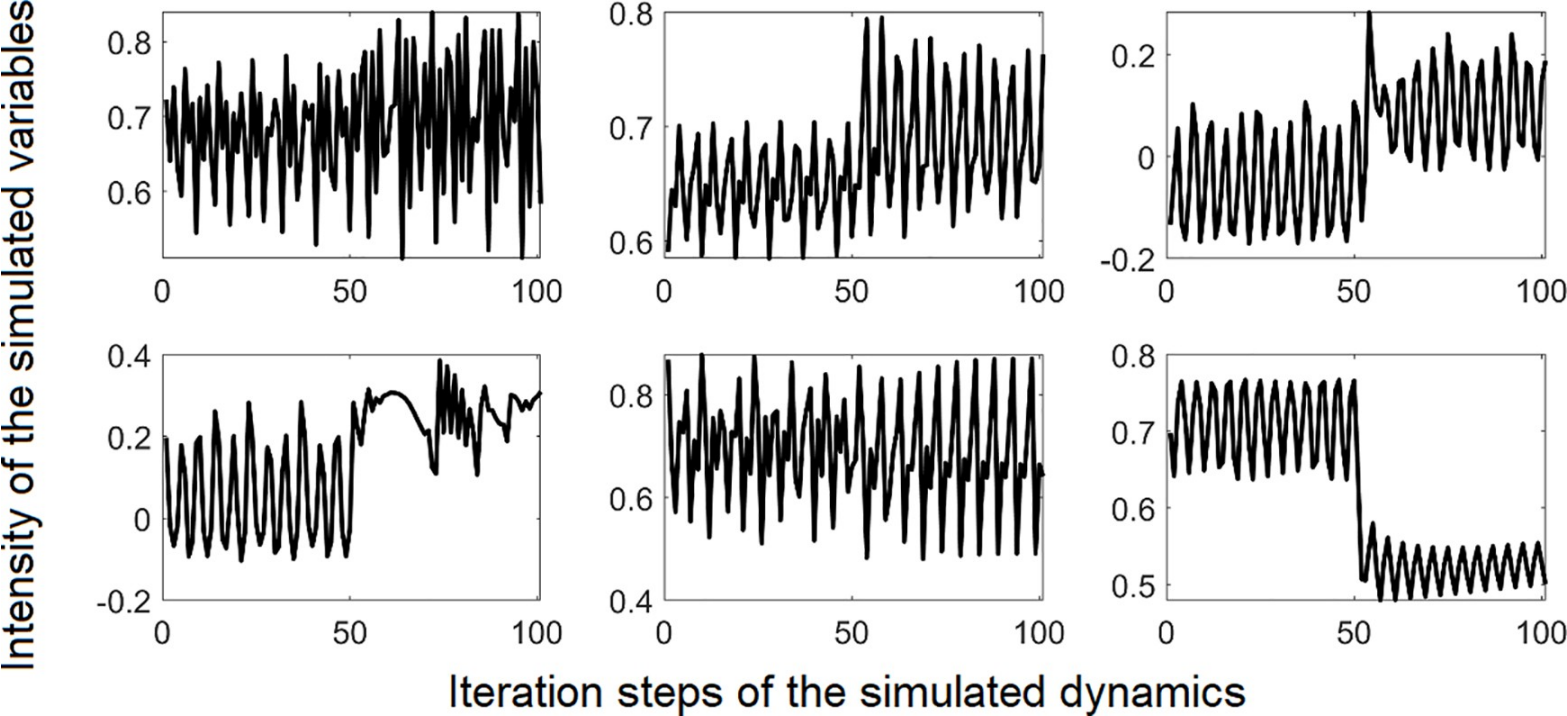
Exemplary time series with pattern transitions that were produced by a model of psychotherapeutic change. The simulated time series were used to optimize and validate the PTDA. Some transitions are characterized by a change of the mean, some by a change of the variance, and others by a change of different features in the domain of frequency or complexity. X-axis: 100 iterations of the simulation runs, corresponding to the segment between iteration 100 and 200 as shown in Fig 2A, lower part. Y-axis: Intensities of the simulated dynamics. The range of the variables produced by the theoretical computational model is [-1, +1] for E, P, S and [0, +1] for I and M.

In terms of Nonlinear Dynamic Systems Theory and Synergetics, the state variables represent the order parameters of the system, and the trait parameters the control parameters [49]. In nonlinear dynamic systems, the control parameters are the relevant entities that need to be changed if a phase transition is to occur. The phase transition can be observed on the level of the order parameters by a change of their dynamic patterns. In the computational model, pattern transitions were simulated by increasing or decreasing the control parameters of the system. Examples are depicted in Fig 1. All time series can be found in the Supplement 1 in S1 File.

#### Generation of the time series

**Fig 2:**
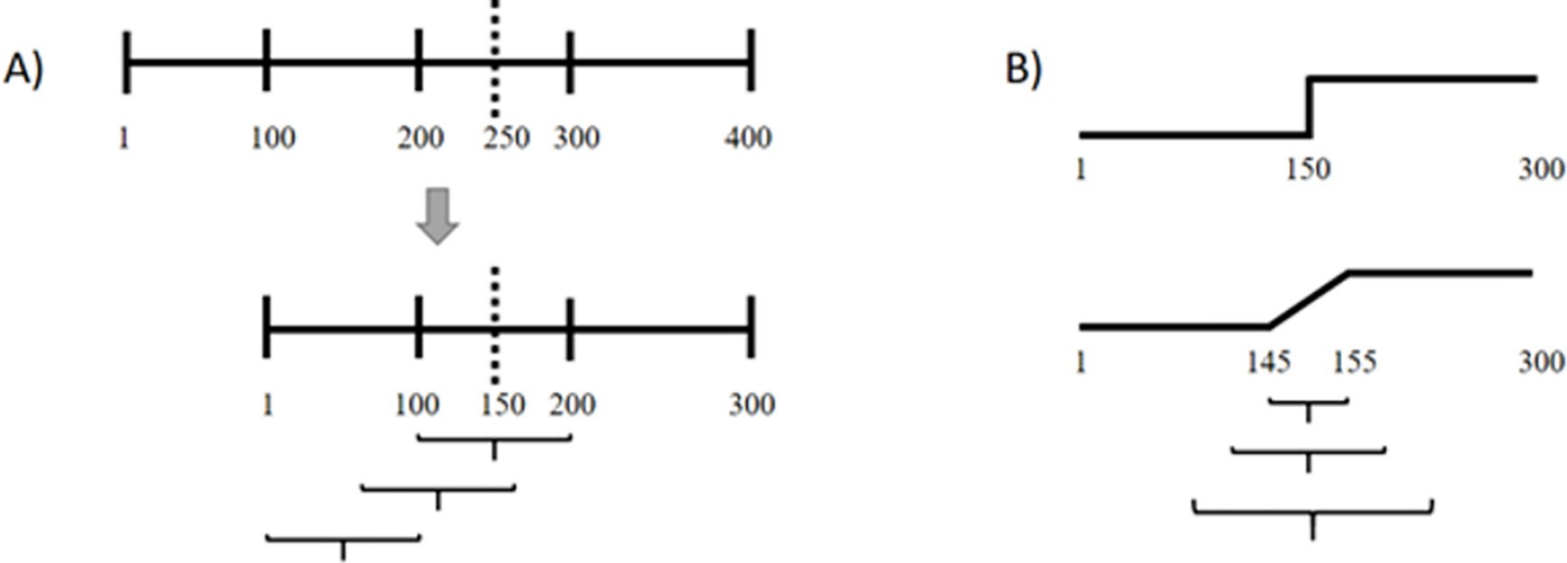
Generation of the time series. **A)** Simulations with 400 iterations (data points) were generated. The first 100 data points were discarded to allow for saturation effects (transient period). A transition point (dashed line) was launched at T = 250 in the original time series, corresponding to T = 150 in the adjusted time series. Depending on where the transition point should be, the corresponding interval of 100 data points (brackets) was chosen and assessed with the algorithm. The upper bracket shows the interval where the transition point is located at the middle of the time series, the bracket in the middle the interval where the transition is at the end, and the lower bracket the interval without a transition point (to assess the rate of false positive results). **B)** The effects of prolonged periods of changes were investigated by linearly increasing the control parameters over several time points (brackets).

Simulation runs of *T* = 400 iterations were generated with random initial values between 0 and 1 for the variables and the parameters. At *T* = 250, a pattern transition was induced by instantaneously increasing all control parameters by a random value between 0 and 1 with upper and lower limits of [0, 1] for the final parameters and a minimum change of +/- 0.05. Each simulation produces 5 time series (one for each of the five variables of the model). The first 100 data points of each time series were discarded to account for saturation effects (transient period); the TP was thus located at *T* = 150 (Fig 2). 100 out of the resulting 300 data points were used depending on where on the time series the TP was evaluated. For example, the interval [100, 200] was used to simulate a transition in the middle of the time series (*T* = 50) (Fig 2A), the interval [70, 170] to simulate a transition at the end of the time series (*T* = 80). To assess the rate of false positive results, the interval [100, 200] was used, i.e., an interval without a transition.

As is common in nonlinear dynamic systems like the computational model of psychotherapy, the dynamics produced by the system can be a fixed point (i.e., the value of the variable stays the same all the time), periodic behavior of different orders (e.g., in a 4-cycle, the same value reoccurs every 4^th^ data point; a pendular would correspond to a 2-cycle), and chaotic behavior, where the same value never occurs again. To mimic empirical time series most appropriately, we only included simulation runs that had sufficiently complex character, i.e., where the resulting time series were either of high periodicity (minimum: 16-cycle) or chaotic. Each simulation run of the theoretical model created 5 time series, which implies that the 60 simulation runs produced a sample of 300 time series.

#### Empirical data

In order compare the application of the PTDA to simulated time series with its application to empirical processes, 4 empirical time series of different psychological variables were assessed with the PTDA to illustrate the applicability on empirical data. One time series (see below, Fig 6A) represents the depression subscale of the SCL 90 [52] from the publicly available dataset of a case study [53]. For these time series data, a change point had already been identified [54], which we try to replicate with the PTDA. Three other time series of psychotherapeutic processes were generated by the Synergetic Navigation System (SNS) [1], an online-based real-time monitoring system, which is routinely used by patients during inpatient and outpatient therapy. In routine practice, daily self-assessments are realized by answering a validated and standardized process questionnaire like the Therapy Process Questionnaire (TPQ, [55]) or a personalized questionnaire which usually is developed together with the patient. One of the empirical time series (Fig 6B) was taken from a case report on a patient with dissociative identity disorder and shows one item of an individualized questionnaire which represents stress-related experiences corresponding to the “child-state” of the patient [3]. Another time series (Fig 6C) shows the daily ratings of the item “experienced therapeutic progress” of a patient (diagnosis: Major Depressive Disorder), assessed by the TPQ. The fourth time series (Fig 6D) represents the dynamics of the factor “motivation for change” of a patient with the diagnosis of Major Depressive Disorder (also assesses by the TPQ). The empirical time series can be found in Supplement 3 in S1 File.

### The algorithm

**Fig 3:**
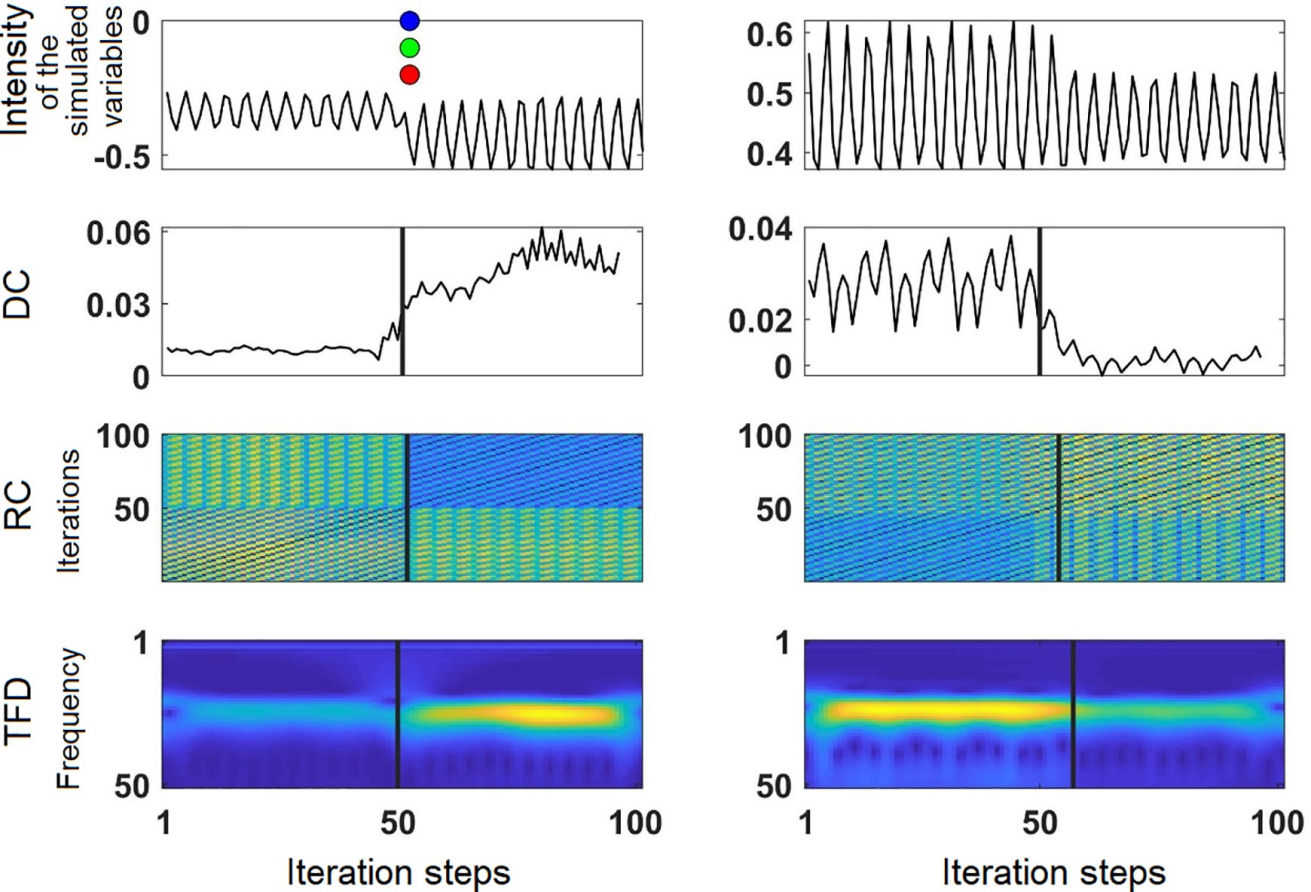
Methods of the PTDA. From top to bottom: original time series, Dynamic Complexity, Recurrence Plot, Time Frequency Distribution. The left column shows an example of a time series where all methods find a transition at a converging point (T = 52). The blue circles show points where a change of the mean was detected, the green circles where a change of the variance was detected, and the red circles where a change of the linear trend was detected by the CPA (in this example, the points are identical; note that the vertical placement of the points is irrelevant and adjusted for visual recognition only). The right column shows an example where no change point was found by the CPA (i.e., applied to the original time series), but where the other methods convergently found a transition point at T = 53. X-axis: Time series length (see Fig 2A for explanation). Y-axis: From top to down: Value range of the simulated time series; values of the Dynamic Complexity measure; rows of the Recurrence Plot (time x time); frequency spectrum of the Time Frequency Distribution.

The original idea of using the combined information of several methods to identify transition points was first presented in [26], which also describes additional methods that might be used. The process of optimizing the algorithm led to the exclusion of two methods: permutation entropy [56] showed unreliable results and depended considerably on the parameters used (word length and window width), and the instantaneous frequency [57] was not sufficiently able to reduce the matrices of the Time Frequency Distribution to a vector. While permutation entropy was excluded without replacement, the Time Frequency Distributions are now assessed in the same way as the matrices of the Recurrence Plots, i.e., by assessing each line separately with the CPA (see below). An example of two time series is shown in Fig 3.

#### Description of the methods used in the PTDA algorithm

*Change Point Analysis (CPA) on original time series and on secondary analyses methods*. The CPA [31] as implemented in the function *findchangepts* in Matlab (R2018b) was applied to the raw time series, and in addition to the results of the secondary analyses methods. The method is sensitive to changes of specific statistical properties of a time series. A time series x contains a change point if it can be split into two segments x1 and x2 such that C(x1) +C(x2) <C(x), where C represents the cost function, here C(x) = Nvar(x), where N is the number of time points of x. In other words, a change point is detected between the segments x1 and x2 of a time series if the sum of the variance of the statistical property of interest, e.g., the mean of the segments, is smaller than the variance of this property of the whole time series; otherwise, no change point is detected.

Consider the time series {2,2,2,4,4,4,4,4,4,4}, where the mean changes from 2 to 4 between t = 3 and t = 4. The change point analysis algorithm first splits the time series into two segments, x1 from t = 1 to t = 2, and x2 from t = 3 to t = 10. For both segments, the cost function C is calculated: the first part includes N = 2 time points, the second part N = 8 time points with var(x1) = 0 and var(x2) = 0.5, hence C(x1) = 2∙0 = 0 and C(x2) = 8∙0.5 = 4. The sum of C(x1) and C(x2), 4, is then compared to the cost function of the whole time series, C(x) = 10∙0.933 = 9.33. Since C(x1) + C(x2) are not less than C(x), the algorithm concludes there is no change point when segmenting the time series after t = 2. It then proceeds by splitting the time series between t = 3 and t = 4 and repeats the tests for these segments. Now, both the variance of x1 and x2 are zero, hence C(x1) +C(x2) <C(x); a change point is detected correctly between t = 3 and t = 4.

The function is able to detect changes of the mean, of the variance, and of the linear trend of a time series. In the PTDA, these three possibilities are used by default for the original time series. For the application on the secondary methods, only changes with respect to the mean are assessed. In principle, the CPA can detect multiple change points in a time series. Also, the PTDA was designed to be applied to numerous transition points but was restricted to a maximum of 1 in the validation study because the simulated time series were designed to have exactly one transition point.

#### Dynamic Complexity (DC)

The Dynamic Complexity measure [24, 38] was developed to identify critical instabilities in short and coarse-grained real-world time series, without further mathematical or parametric assumptions. DC mirrors the increased complexity and sensitivity to noise and perturbations of system dynamics before phase transitions, but also the fact that regimes or attractors of human dynamics realize different degrees of complexity. DC is the multiplicative product of a fluctuation measure and a distribution measure applied to discrete time series data with given data ranges and constant discrete time intervals between the data points. The fluctuation measure (F) is sensitive to the amplitudes and frequencies of a time signal, and the distribution measure (D) scans the scattering of values or system states occurring within the range of possible values or system states. In order to identify non-stationarity, DC is calculated within a data window moving over the time series (sliding window). Because the empirical time series were collected by daily ratings, we apply a window width of 7 measurement points (corresponds to one week). The window size can be adjusted by the user in the Matlab version of the PTDA, but the effect of different window sizes on the performance of the algorithm was negligible. For the PTDA, the Matlab Toolbox to calculate the DC by Viol [58] was used. The DC is also implemented in the SNS. A detailed example how to calculate Dynamic Complexity is given in the Supplement 4 in S1 File.

#### Recurrence Plots (RP)

Recurrence Plots provide a visualization and quantification of recurrent, i.e. dynamically similar states within a time series [40, 59]. Dynamic similarity is measured in terms of some metric distances defined in the underlying state space. One or more time series are projected into a multidimensional state space by embedding procedures with a specific embedding dimension m and a time-delay parameter τ. The method of time-delayed embedding allows the reconstruction of phase-space profiles from a single one-dimensional time series, following the logic of Taken’s theorem [60] but also from multi-dimensional time series [42–44, 61]. Dynamic similarity is measured in terms of metric distances $d_{ij}=\|\vec{x_{i}}-\vec{x_{j}}\|$ defined in the system’s reconstructed state space. Usually Recurrence Plots are produced by binary matrices where an entry is 1 if *d*_i,j_, ≤ ε, otherwise it is 0 (where x_i_ and x_j_ are elements of the time series and ε some threshold). This procedure depends on three parameters: the embedding dimension m, the time delay τ for taking elements of the time series x_i_ and the threshold ε which defines the occurrence of recurrent (close) or distant (non-recurrent) state vectors in the m-dimensional embedding space.

In the PTDA we selected a small embedding dimension of m = 3 and a small time delay (τ = 1) which preserves the biggest number of state vectors out of a given time series. In psychotherapy we are frequently challenged by the availability of only short time series, e.g. 60 or less session by session measures or daily measures in a hospital stay. Instead of defining a specific threshold distance ε we used the Euclidian distance between all vector points $\vec{x}$ in the time delay embedding space. In consequence the recurrence matrix **R** is equivalent to the distance matrix **d** = (*d*_i,j_). In the time x time recurrence matrix, the color of each entry reflects the dynamic similarity between all vector points, with dark blue representing identity or very short Euclidian distances between the vector points and red representing the longest Euclidian distance between any two vector points (rainbow color spectrum). In the PTDA, the recurrence plots are calculated by a Matlab Toolbox [62, 63] with the commonly used parameters m = 3 and *τ* = 1. Note that during the optimization of the algorithm, the results were largely unaffected by the choice of these parameters. Of course, they can still be changed by the user in the Matlab version of the PTDA.

#### Time frequency distribution (TFD)

Time frequency distribution (TFD) is a method to calculate and visualize the frequency of a signal (time series) as it changes with time [64, 65]. In order to identify frequency changes, a moving window approach is implemented. Mathematically, both time t and frequency ω are variables of a distribution P(t,ω) which describes the amplitude (energy) of the signal at each given t and ω. Here, we use the so-called Stockwell transform (S-transform) which is a combination of two common TFD-methods, the Short Time Fourier Transform and the Continuous Wavelet Transform [37]. It preserves the phase information available from the former method but uses the variable (i.e., not fixed) window length of the continuous wavelet method. For visualization, time and frequency are plotted on a plane (x: time, y: frequency) and color coding is used for the representation of the amplitude (energy) of the frequencies. In the PTDA, a Matlab Toolbox by Sundar [66] is used.

#### Steps of the algorithm

**Fig 4:**
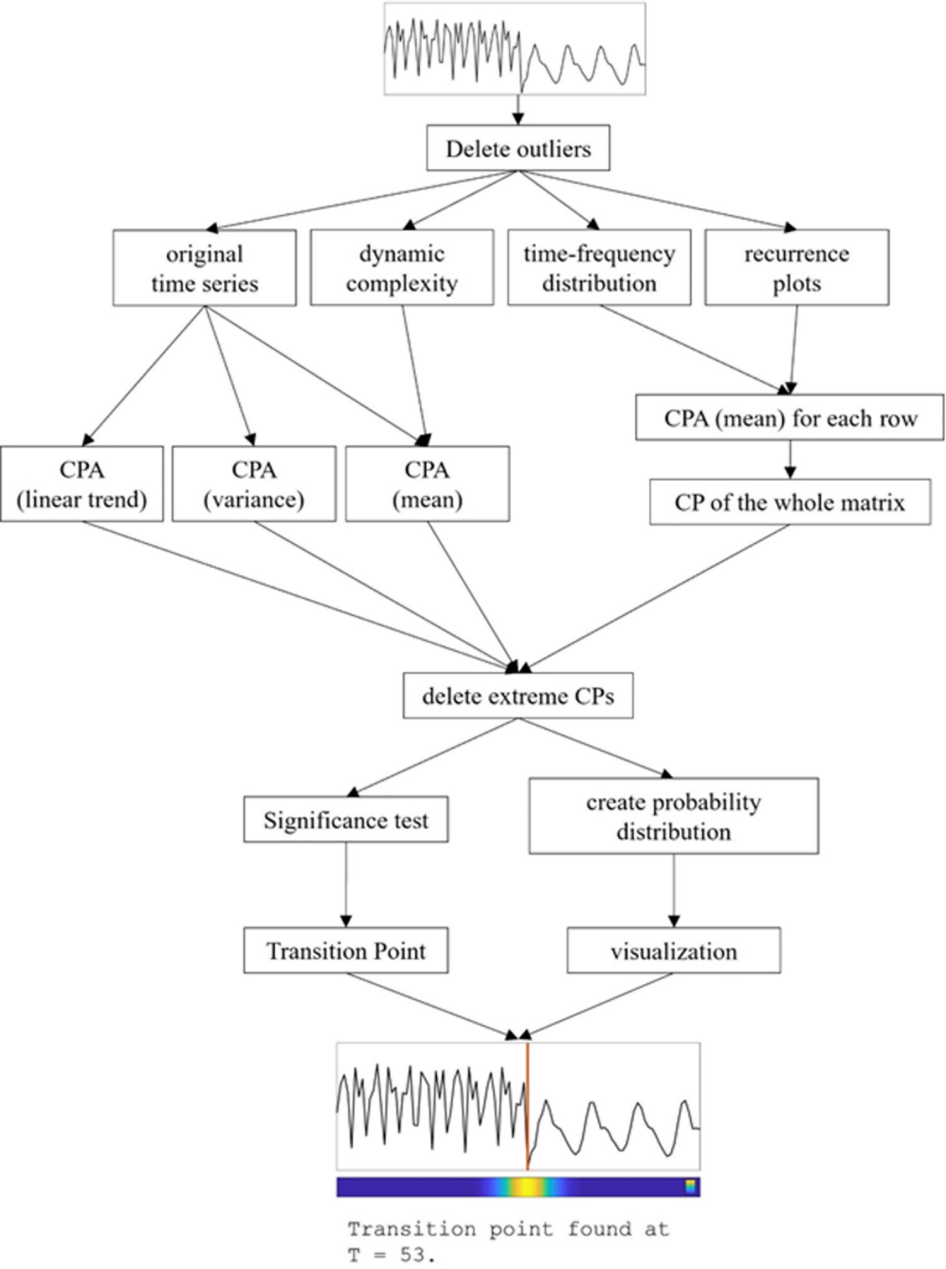
Flow chart of the Pattern Transition Detection Algorithm (PTDA). CP: change point; CPA: change point analysis.

*Outlier deletion*. For each time series, the following steps are performed by the algorithm (Fig 4): First, outliers are deleted with the Matlab function *isoutlier* with option *‘gesd’*. The function applies an iterative algorithm to determine outliers based on the Generalized Extreme Studentized Deviate test (MathWorks, 2020).

#### Application of secondary methods

Second, the secondary methods (Dynamic Complexity, Recurrence Plot, Time Frequency Distribution) that were described in detail above are calculated. This results in a total of two time series (the original one plus the one of the Dynamic Complexity) and two matrices (Recurrence Plot and Time Frequency Distribution).

#### CPA

For the original time series and the DC, the CPA can be directly applied. This results in a maximum of four change points: one each for changes of the mean, the variance, and the linear trend of the original time series, and one for a change of the mean of the Dynamic Complexity. Note that it is possible that fewer change points are found if one or more methods do not find a change point. For the matrices (Recurrence Plots and Time Frequency Distributions), the CPA with respect to the changes of the mean is applied to every line. Then, a histogram of the change points of all lines is build, and the peak of the histogram determines the point of change for the whole matrix. In detail, the bin width of the histogram is fixed to (1/5)^th^ of the length of the time series and the “peak” of the histogram is the (rounded) middle of the bin containing the most values.

#### Deletion of extreme CPs

An observation from inspecting false-positive results from the CPA of numerous time series was that these were nearly almost located at the very extremes of the time series. One important step in reducing the rate of false-positive results was therefore the exclusion of TPs that were found within the first or last 10% of the length of the time series. For applications in psychotherapy research, this also makes sense from both a psychological as well as a dynamical systems theory point of view. It is well known [24, 67] that the beginning and the end of psychotherapy are specific periods with increased fluctuations due to changes of the setting. These changes are, however, not what we want to detect in psychotherapy research. This is backed up by dynamic systems theory, which describes how changes in the boundary conditions (the setting) lead to a transient period until settling at a stable attractor [24].

#### Derivation of the overall TP

For each matrix as well as for the different methods, several change points will be found. In the optimal case, all change points will be at the same position on the time series and result in a clear overall transition point (TP). This, however, is a very rare case and hardly ever happens in time series with complex patterns. The main part of the PTDA will therefore assess the different change points in order to derive the most valid and reliable point of transition. The steps to achieve this are described in the following.

#### Significance testing

As described in [26], a valid TP should be characterized by a clustering of CPs around a certain point (the real TP) of the time series. This dispersion along the time series should be different to the dispersion of points that are placed randomly on the time series. Inspired by bootstrapping, random values were drawn from a discrete uniform distribution of the length of the respective time series with the *unidrnd* function implemented in Matlab. The number of random values drawn is equivalent to the number of change points found by the different methods for the respective time series. This is repeated 100 times. The spreading of the points of non-normal distributions can be assessed by the interquartile range (IQR). The IQR describes the number of points within the second and third quartile of the data, i.e., the inner 50% around the median, and is a measure of the dispersion of the data comparable to the variance of normally distributed data. After calculating the IQR of the 100 sets of random CPs, the mean and the 95% confidence interval of these IQRs is calculated. If the IQR of the real data lies below the lower bound of the confidence interval of the equally distributed random data, the TP is considered significant.

Importantly, the algorithm does not test if the mean (or any higher order moment) is significantly different before and after the change point. If required by the research question, this can easily be calculated afterwards once the transition point is known.

#### Construction of the probability distribution

The last step of the algorithm consists of generating probability distributions in order to gain a visualization of the overall result. For each change point found by the different methods, a normal distribution is constructed where the mean is the position of the change point and the variance is fixed at 5, what is an arbitrary but very restrictive threshold. The height of the distribution is normalized to one. Then, the sum of all normal distributions is calculated and visualized as a color band where high values are shown in yellow and low values in blue. This kind of visualization provides an intuitive impression about how prominent the transition point is in terms of convergence among change points: points with a high convergence of change points will show as narrow yellow areas in the probability band, while less “clear” transition points will result in broader greenish areas in the band. In addition, this visualization provides important information about possible other transition points, i.e., if there might be more than one transition in the time series.

#### Investigation of the whole system

The PTDA allows as input either single time series, or the time series of different variables of the system together (multiple time series). If more than one time series is entered, the PTDA also provides an overall TP and calculates the significance test and the overall probability function by using the CPs of all methods and time series. Here, the 5 time series generated by each simulation run were assessed as single time series as well as in combination.

### Assessment of the performance

Five measures are calculated to quantify the different aspects of performance of the PTDA and the CPA: mean, standard deviation (SD), precision, rate of false negatives, and rate of false positives.

#### Mean and SD

The mean and SD of the transition points (TP) of all time series. The mean should, ideally, be the point of the real transition (e.g., at 50 for a transition in the middle of the time series with 100 time points). The standard deviation should be as small as possible.

#### Precision

To assess the precision of the algorithm, we calculated the rate of transition points (TPs) that were within +/- 5 time points around the real TP. Ideally, all TPs should lie within this interval.

#### False negatives

If the algorithm does not find a TP although there is one, this is counted as a false negative result. The rate of false negative results should be below 5%.

#### False positives

To assess the rate of false positive results, the first 100 time points of each simulated time series, where no transition occurred, were used (see Fig 2A). This guarantees that this measure is not influenced by a specific pattern, since these time series had the same pattern as the ones used to assess the transitions. The rate of false positive results should be below 5%.

### Modeling real-world conditions

**Fig 5:**
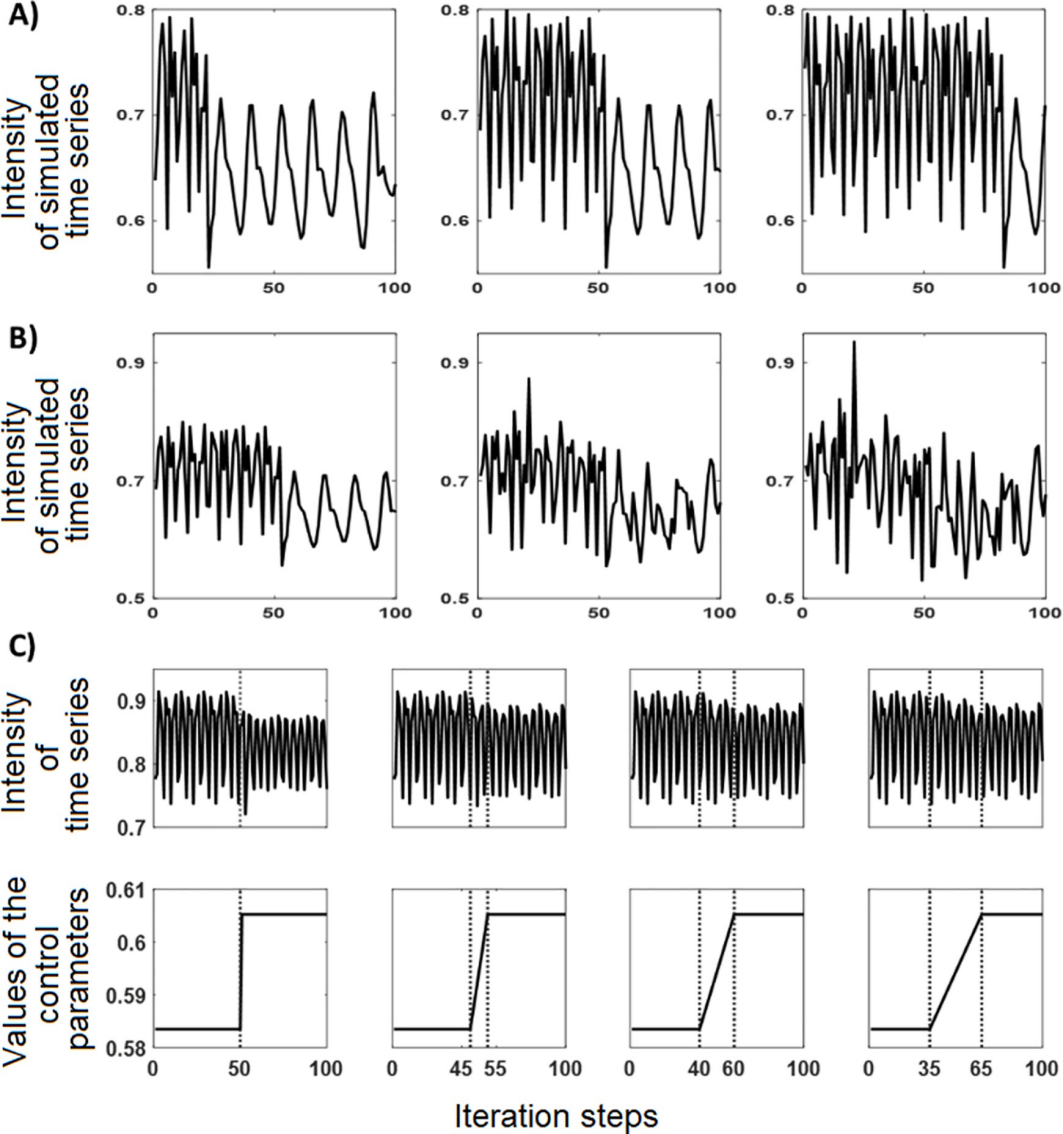
**A)** Different positions of the simulated transition point (TP) at T = 20 (left), T = 50 (middle) and T = 80 (right). **B)** Different levels of noise were added to the original time series in order to investigate the effect on the PTDA. Left: Original time series without noise (deterministic). Middle: original time series with noise with a variance of +50% of the variance of the original time series. Right: original time series with noise with a variance of +90% of the variance of the original time series. **C)** Prolonged transitions. The upper rows show the time series resulting from the parameter increases (lower row). Left: original time series with a sudden pattern transition induced by an instantaneous increase of the control parameter from one to the next iteration. Middle and right: Time series and linear parameter increases in intervals of 10, 20, and 30 iterations (time points). X-axis: time series length. Y-axis: Rage of the time series values. C lower row: drift of parameter values.

Furthermore, the performance was tested under conditions comparable to empirical time series: noise, shifted, and prolonged transition points. Shifted TPs were assessed by assessing different parts of the time series (Fig 2A). TPs at T = [20, 30, 40, 50, 60, 70, 80] were investigated. Prolonged periods of transition were simulated by linearly increasing the control parameter (Fig 2B) for intervals of 10, 20, and 30 time points. Examples of resulting time series are shown in Fig 5.

## Results

### Performance of CPA and PTDA

The aim of our study was to assess the performance of the PTDA, an expansion of the CPA, in short time series with complex pattern changes as they are found in psychotherapy processes. Five criteria of performance were investigated: first, the mean TP found over all 300 time series used for the validation study; second, the standard deviation of the mean TP; third, the precision defined as the percentage of time series where the TP found by the algorithm within ± 5 time points around the real TP; fourth, the rate of false negative results, calculated as the percentage of time series where no TP was found although there was one; and fifth, the rate of false-positive results, calculated as the percentage of time series where a TP was found although there was none.

As can be seen from Table 1, both algorithms are, on average, able to find the position of the change point correctly: the real change was induced by *T* = 50 in the simulated time series, and the change point was found at 51.4 for the PTDA when all 5 time series generated by the system are taken into account simultaneously, and at 51.7 if the time series generated by the system were investigated independently of each other. Note that it takes some time (some iterations) before a shift of the parameters “arrives” in the system. The % of time series that were within a window of +/- 5 time points around the real transition point was slightly higher for the PTDA compared to the common CPA, and 100% if the time series of the system are assessed together.

**Table 1:** Comparison of the PTDA when (A) the 5 five time series per simulation are assessed simultaneously, i.e., the whole system is taken into consideration, when (B) the time series are assessed independently, and (C) when the common CPA algorithm (with respect to the change of the mean) is applied to the single time series. Based on the 300 simulated time series from 60 simulation runs with an induced change at T = 50, the table shows the mean change point of the algorithms, the standard deviation (SD), the precision, the false negative rate, and the false positive rate. Both algorithms are able to correctly detect the TP in most cases, but the percentages of false negative and false positive findings are much lower in the PTDA.

	Algorithm	Mean	SD	Precision +/- 5	False negatives	False positives
A)	PTDA (whole system)	51.4	1.4	100%	0%	4%
B)	PTDA	51.7	3.3	92%	1%	1%
C)	CPA	52.1	2.1	83%	16%	19%

The main improvement of the PTDA are the rates of false positive and false negative results. The common CPA indicated no change when in fact there is one in 16% of the cases (false negatives), and indicated a change when in fact there was none in 19% of the cases (false positives). In contrast, the rates of false positive and false negative results were below 5% for the PTDA.

### Performance under real-world conditions

#### Shifted transition point

In empirical time series, the transition will often not be in the middle of the time series but shifted towards the beginning or the end. We therefore investigated how a change point at a different position affects the results. The results shown in Table 2 indicate that the algorithm is well able to identify transition points also at other points, and the rate of false negative results is hardly affected.

**Table 2:** Results of the performance of the PTDA under three real-world conditions. *Noise*: different levels of noise were added to the original time series. The numbers in the second column denote the strength of noise, e.g., 50 refers to added noise with a variance of 50% of the original time series. Position: The transition point was shifted along the time series. The numbers in the second column denote where the change was induced, e.g., 50 refers to a transition point at 50% (the middle) of the original time series. Increase: The transition was not induced abruptly but over a longer period of time. The numbers in the second column denote the range of the time series where the change occurred, e.g., 20 denotes that the change stretched over 20% of the time series (see also Fig 5).

		all time series of the system				single time series of the system			
		M	SD	Precision (%)	False neg. (%)	M	SD	Precision (%)	False neg. (%)
Noise	0	51	1.4	100	0	52	3.3	92	1
	10	51	1.4	100	0	52	3.2	92	1
	20	51	1.4	100	0	52	3.4	90	1
	30	51	3.0	98	0	52	4.0	90	1
	40	51	2.9	98	0	51	4.5	85	1
	50	51	5.0	95	0	51	6.0	82	2
	60	51	4.9	95	0	51	7.4	77	2
	70	51	5.1	95	0	50	8.5	70	4
	80	51	5.5	93	0	49	9.8	65	6
	90	50	6.4	85	0	48	10.0	63	7
Position	20	22	2.1	98	0	22	5.9	90	2
	30	31	1.2	100	0	32	2.8	91	3
	40	41	1.3	100	0	42	3.5	91	1
	50	51	1.4	100	0	52	3.3	92	1
	60	61	1.2	100	0	62	3.1	94	1
	70	71	1.2	100	0	71	2.5	93	2
	80	81	1.1	100	0	81	1.8	96	3
Increase	0	51	1.4	100	0	52	3.3	92	1
	10	51	1.7	98	0	51	4.3	91	8
	20	51	2.4	95	0	51	4.7	90	10
	30	51	4.5	87	0	51	5.0	88	12

False neg: Number of false negative results in %; Precision: the detected transition point was within +/- 5% of the real change point.

#### Noise

Although we already used complex time series for validation, a systematic evaluation how noise affects the performance of the PTDA algorithm can shed further light on its applicability. As expected, decreased signal-to-noise ratios reduced the precision (Table 2). When all time series of the system are taken into consideration, the precision remained above 80% even for high levels of noise. Even for very high levels of noise, the rate of false negatives did not rise above 7%, indicating a good performance even in the presence of noise. The precision decreased slightly but dropped below 80% only at noise levels with a variance of >50% of the original time series.

#### Prolonged transition

In practice, the traits of a client (control parameters of the model system, [49]) will usually not change abruptly. Skills and resources, cognitive competencies, hopefulness/trait motivation, and alliance might build up gradually during psychotherapy. In the simulation, this is reflected by a continuous shift of the parameters over an extended period of time. The performance of the algorithm was tested for linear increases over 10, 20, and 30 time points. Since the linear increase of the control parameter did not (in contrast to classical physical synergetic systems) lead to an abrupt change in the pattern, but rather a smooth transition period. In consequence, the performance of the PTDA dropped with increasing length of the interval.

### Minimum length of time series

For practical applications, it is important to know how long a time series must be in order to apply a method like the PTDA. The algorithm was tested on shorter sections of the time series with the TP placed in the middle. The results (Table 3) of the performance indicators are given for time series between 20 and 100 time points. Interestingly, the correct identification is not affected by the length of the time series, as indicated by a mean very close to the real TP, a constantly small standard deviation, and a constantly high rate of precision. What is affected, however, is the rate of false positive results. When applying the criterion of allowing maximally 5% of such false results, the minimum length of the time series has to be 60 points. In cases where 6% are acceptable, the length could be reduced to 50 points. Below that, the application of the PTDA is not recommended.

**Table 3:** Results of the PTDA for time series (TS) of 20 to 100 time points. The mean (M) and the standard deviation (SD) of the transition points (TP) of the 300 simulated time series are given along with further indicators of the performance: the precision (Prec.), which gives the rate of identifying a TP within +/- 5 time points around the real TP, and the rates of false negative (FN) and false positive (FP) results.

Length of TS	real TP	*M*	*SD*	Prec.	FN	FP
100	50	52	3.3	92%	1%	0%
90	45	48	3.5	91%	1%	0%
80	40	43	4.0	91%	1%	0%
70	35	38	3.2	90%	3%	0%
60	30	33	3.2	89%	3%	0%
50	25	27	2.6	90%	3%	6%
40	20	22	2.5	87%	5%	13%
30	15	17	2.4	89%	6%	19%
20	10	11	1.9	88%	11%	44%

### Empirical examples

**Fig 6:**
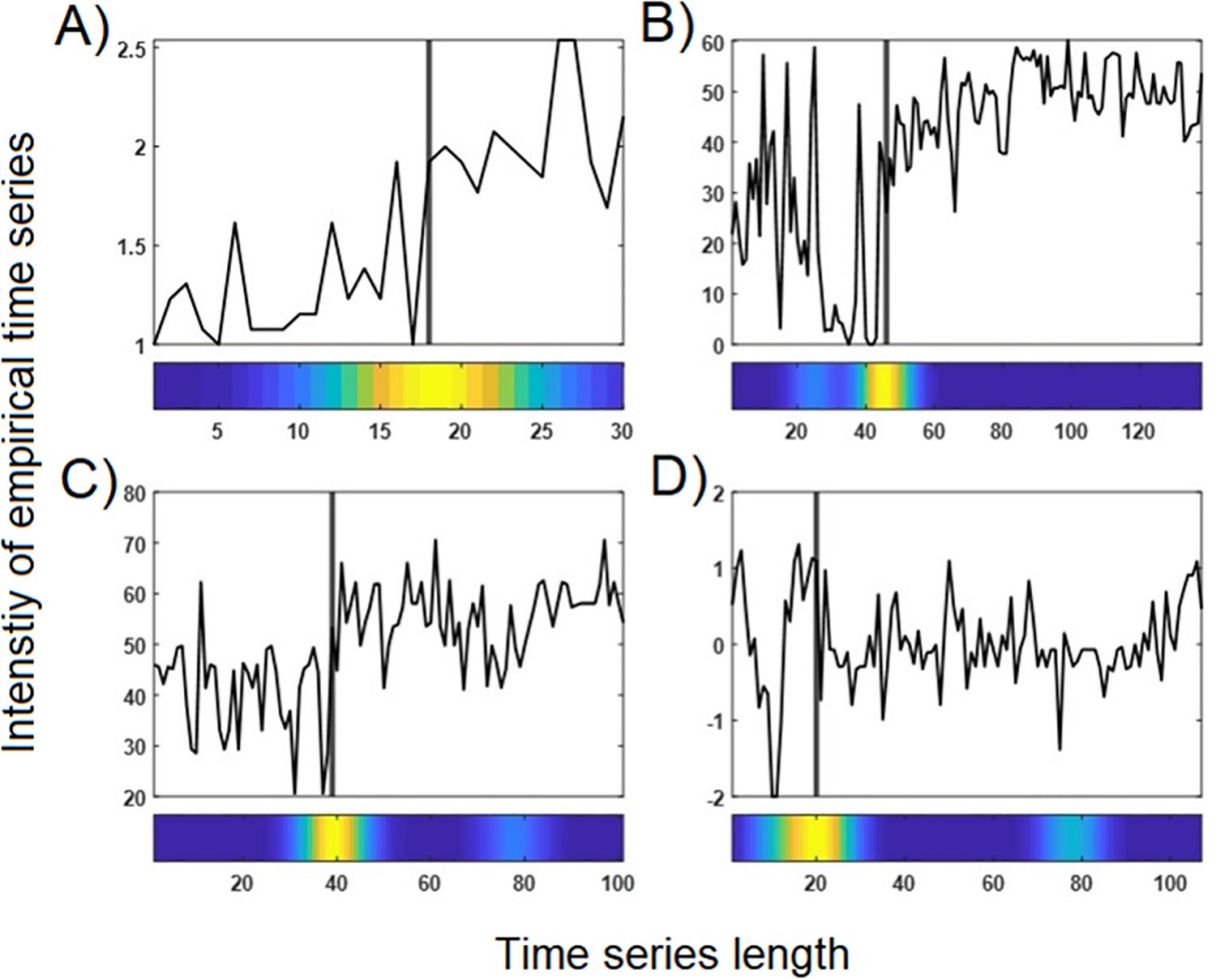
PTDA applied to four different psychological time series. A) Time Series of the depression subscore of the SCL-90 from a open access dataset (see Methods). B) Time series of the “child-state” of a patient with dissociative identity disorder during the psychotherapy process (Schiepek et al., 2016). C and D) Time series of the psychotherapeutic process. The change point is marked by the vertical black line. The bar below gives an impression of the convergence of the CPs over all methods. X-axis: Measurement points (daily assessments): Y-axis: Intensities of the values (A, B, C); z-transformed values of the factor “motivation for change” (D).

Finally, the PTDA was tested on several empirical time series (see Methods). The output of the PTDA for these time series is shown in Fig 6. The examples reveal that the TPs are found at positions where one would expect them by visual inspection of the time series. For Fig 6A, the transition is less clear than in the other examples, as can be seen by the broader yellow area in the probability band below.

#### For comparison

The common CPA with the criteria mean (M), standard deviation (SD), and linear trend (LT) was applied to the 4 empirical time series as shown in Fig 6. In (A): (M) 18, (SD) no result, (LT) 18, (PTDA) 18. In (B): (M) 49, (SD) no result, (LT) 44, (PTDA) 46. In (C): (M) 39, (SD) no result, (LT) 39, (PTDA) 39. In (D): (M) no result, (SD) no result, (LT) 12, (PTDA) 20. Whereas the results from common CPA and PTDA are identical in (A) and (C) or similar in (B), in one of the empirical time series (D) the methods identified different TPs (based on CPA(LT) only). In no case the CPA for standard deviations could identify a TP.

### Comparing PTDA with qRQA

A rapidly growing state-of-the-art branch of the Recurrence Plot method, the Recurrence Quantification Analysis (RQA), represents a powerful toolbox for processing nonstationary and noisy time series. Recurrence-based techniques were developed around their power of capturing the dynamics of complex and non-stationary time series data and of time series exhibiting qualitatively different patterns along with their temporal evolution [39, 40]. RQA introduces various metrics to quantify data complexity from different angles and at different time scales. RQA quantifiers allow efficient detecting of transitions, e.g., in chaotic model systems [61] and electrophysiological data [41].

**Fig 7:**
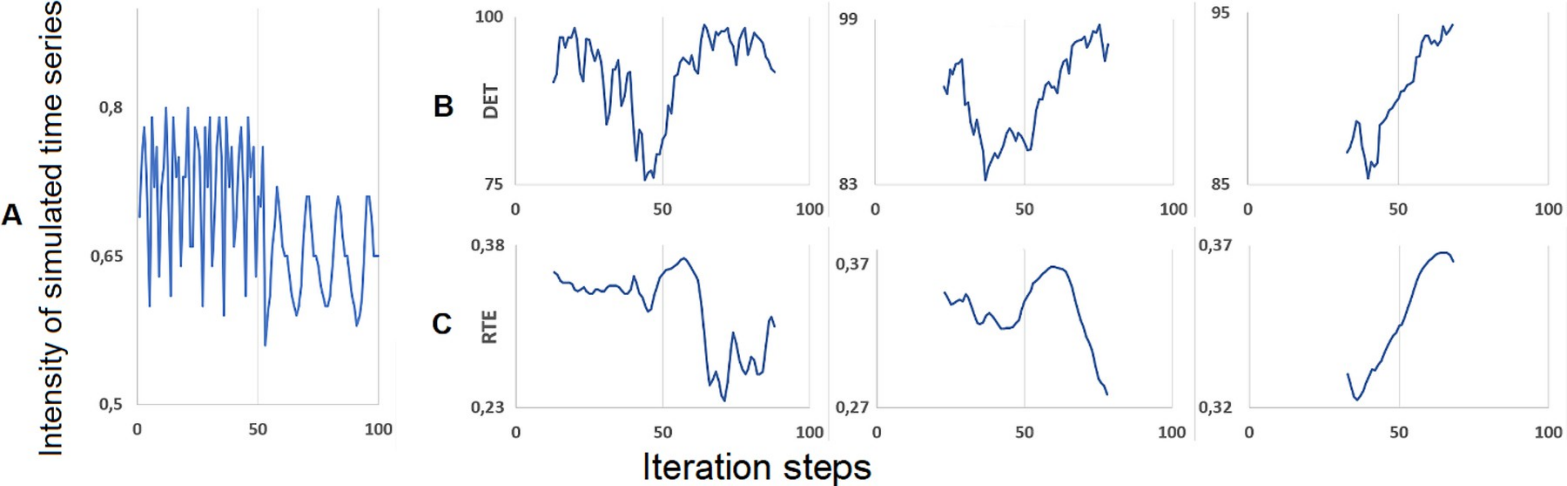
Evolution of DET (Determinism) and RTE (Recurrence Time Entropy) applied to a simulated time series from our validation set. (A) The time series. (B) Evolution of DET depending on the width of the sliding window (left: 25, middle: 45, right: 65). (C) Evolution of the RET depending on the width of the sliding window (left: 25, middle: 45, right: 65).

For illustrating the method, we applied two different RQA quantifiers on a simulated time series where the point of parameter change was placed at 50 and transition point as identified by the PTDA was at 53 (comp. Figs 4 and 5A, middle). Following [41], the RQA quantifiers we used is the Determinism (DET) which is defined as the ratio of recurrence points that form diagonal lines compared to all considered recurrence points and the Recurrence Time Entropy (RTE) which is an entropy measure based on recurrent vertical lines *t*_w_ in the sliding window.

$$DET=\frac{\sum_{l=l_{min}}^{w}lP(l)}{\sum_{l=1}^{w}lP(l)}$$

with *l*_min_ = 2, *w* is the window width of the sliding window and *P(l)* is the histogram of lines in parallel to the diagonal in the sliding window.

$$RTE=⎼\sum_{t_{w}=1}^{T_{max}}p(t_{w})ln\;p(t_{w})$$

with *t*_*w*_ is the recurrence points in the vertical lines of the sliding window, *T*_*w*_ equals to the number of vertical lines in the window, and *p(t*_*w*_*)* is the estimated probability of recurrence points in the vertical lines, corresponding to the recurrence rate. The parameters we used for construction the Recurrence Plots are m = 3 embedding dimensions, delay τ = 1, and threshold ε = 0.1. The calculation was done with the crqa package in R [39]. Fig 7 shows the results.

Depending on the window width, the DET quantifier increases when the signal gets more regular and rhythmic whereas the RTE decreases. This corresponds to the anti-correlated behavior of both measures as shown in [41] for movement-related EEG data. During the pattern transition (shortly before or behind), DET decreases and RTE increases which may mirror the increased complexity of the time series during the transition. Evidently, the exact position of the changes in DET and RTE depends on the window width of the submatrix of the Recurrence Plot which was used to calculate the quantifiers. The broader the window, less the ratio of the complete time series which can be exploited for calculation and the fuzzier will be the result. More than this the evolution of the RTE seems to be inverted (increasing), as the broadest sliding window only captures the increasing segment of the dynamic measure (see Fig 7C, left and middle).

Based on this illustrative case, it became evident that RQA quantifiers are able to identify nonstationary transitions in complex systems. The preciseness will depend on the time series length, with impressively good results for longer time series, as in movement-related EEG signals [41] or in simulated time series moving into or out of chaotic regimes [61]. In some fields of application, like psychotherapy, the PTDA could be a useful amendment and complementary method to RQA. A more systematic cross validation of PTDA and RQA quantifier is necessary and will be prepared by the authors.

## Discussion

An expansion of the CPA algorithm, the Pattern Transition Detection Algorithm (PTDA), was evaluated with 300 simulated time series with known transition points. The PTDA combines the information from three nonlinear methods: Recurrence Plots show changes of repeating patterns and in this sense are linked to autocorrelation, Time Frequency Distributions depict changing frequencies over time, and Dynamic Complexity captures the dynamics of fluctuation, amplitude, and distribution of a time series. Assessing all these aspects in one algorithm, the PTDA provides an easy-to-use tool for research and practice to define the transition points in any psychological time series that exceeds 50 time points. In contrast to applying the commonly used CPA only, the PTDA is able to detect not only changes of the mean, but also of other important qualities of time series and pattern transitions in general. This allows to reduce the high rate of false negative and false positive results of the CPA to below 5%, while keeping the high precision of the CPA. The small standard deviation further confirms the high precision of the PTDA.

The numerous applications of the algorithm for research are obvious. Whenever one is interested in discontinuous changes within a time series, the PTDA will objectively specify the point of change. Several examples of studies in various fields of psychology have been mentioned in the Introduction.

### Recurrence Quantification of Transitions (RQT)

Another important contribution of this study is the validation of a method to determine the point of change in Recurrence Plots (Recurrence Quantification of Transitions, RQT), which was introduced in [26]. In our study, the calculation of change points of each row in the RPs, which we call Recurrence Quantification of Transitions (RQT), provided reliable and valid results. Sophisticated methods exist to characterize Recurrence Plots as a whole (RQA). In the fast growing field of RQA, new quantifiers were developed in order to identify nonstationary developments and pattern transitions in complex systems. In consequence, PTDA and RQA algorithms could be combined and used for cross-validation, like in complexity science different algorithms for complexity or entropy quantification are applied in combination.

### Diversity of pattern transitions

In practice it is important to see that pattern transitions not only concern changes of the mean level of psychological measures. Usually, the General Linear Model of psychological statistics focuses the attention of psychotherapists on increasing or decreasing levels of symptom intensity, emotions, or experienced progress. In psychotherapy research this is mirrored by the investigation of sudden gains or sudden losses (e.g., [11]). However, there are many examples of changes which concern the frequency patterns and the dynamic complexity of symptoms and emotions, e.g. low vs. rapid cycling of emotions in bipolar disorders, intensity of emotional fluctuations in Borderline Personality Disorder, switching between ego-states which are enslaving different emotions and cognitions in dissociative identity disorders vs. dissolving pathological over-synchronization [3], to be stuck in reduced ways of mental functioning (e.g. craving, numbness) vs. flexibility and adaptivity [68]. If mental disorders can be seen as “dynamic diseases” [69, 70] then we should open our judgment on therapeutic effects on changing dynamics. This will also concern the discussion on the appropriateness of outcome measures in psychotherapy.

### Limitations and future developments

One limitation of our study is that the simulated time series were all produced by one system model. Although the model has been shown to reproduce the dynamics found in empirical time series of the psychotherapy process [49, 50], we cannot exclude that not all features of empirical time series are incorporated. However, the algorithm was shown to work also for the well-known Hénon map [26], one of the best investigated models in dynamic systems research, and worked well with different empirical time series.

In principle, the PTDA can be applied to time series of any kind of any discipline, but the performance was not tested here. Note that for very long time series: while the result is presented at 1.3 seconds for a time series of *N* = 100 data points, it takes 82.2 seconds for *N* = 500 data points and over 7 minutes for *N* = 1000 data points. The running time is mainly affected by calling the function *findchangepts* more than 2*N times in the evaluation of the RP and TFD matrices. The evaluation of speed was done on an a notebook with intel core i7 processor with 16 GB RAM.

Last but not least, the PTDA has been tested and validated for simulated data with one change point. Since the algorithm offers the possibility to find several TPs, the performance on such time series should be evaluated in the future. The main challenge here will be to avoid having to pre-define the maximum number of changes, which is a non-trivial problem in cluster analysis algorithms.

## Supporting information

S1 File Supplement. (PDF)

S1 Data Empirical data. (CSV)

S2 Data Simulation data. (CSV)

## References

[1] Schiepek G, Aichhorn W, Schoeller H. Monitoring change dynamics–a nonlinear approach to psychotherapy feedback. Chaos Complex Lett 2018;11(3): 355–375.

[2] Schiepek G. Aichhorn W, Gruber M, Strunk G, Bachler E, Aas B. Real-time monitoring of psychotherapeutic processes: Concept and compliance. Front Psychol 2016;7(5):604. doi: 10.3389/fpsyg.2016.00604 27199837 PMC4853656

[3] Schiepek G, Stöger-Schmidinger B, Aichhorn W, Schöller H, Aas B. Systemic case formulation, individualized process monitoring, and state dynamics in a case of dissociative identity disorder. Front Psychol 2016;7:1545. doi: 10.3389/fpsyg.2016.01545 27812338 PMC5072376

[4] Lyby MS, Wallot S, Mehlsen MY. Measures of microgenetic changes in emotion regulation strategies across life transitions. In: Viol K, Schoeller H, Aichhorn W, editors. Selbstorganisation–ein Paradigma für die Humanwissenschaften. Wiesbaden: Springer; 2020. pp. 287–302. 10.1007/978-3-658-29906-4_16

[5] Molenaar PCM, Sinclair KO, Rovine MJ, Ram N, Corneal SE. Analyzing developmental processes on an individual level using nonstationary time series modeling. Develop Psychol 2009;45(1): 260–271. 10.1037/a0014170 19210007

[6] Gennaro A, Kipp S, Viol K, de Felice G, Andreassi S, Aichhorn W, et al. A phase transition of the unconscious: Automated text analysis of dreams in psychoanalytic psychotherapy. Front Psychol 2020;11(1667). doi: 10.3389/fpsyg.2020.01667 32903443 PMC7434971

[7] de Felice G, Giuliani A, Gelo OCG, Mergenthaler E, de Smet MM, Meganck R, et al. What differentiates poor- and good-outcome psychotherapy? A statistical-mechanics-inspired approach to psychotherapy research. Part two: Network analyses. Front Psychol 2020;11(788). doi: 10.3389/fpsyg.2020.00788 32508701 PMC7251305

[8] Olthof M, Hasselman F, Strunk G, Aas B, Schiepek G, Lichtwarck-Aschoff A. Destabilization in self-ratings of the psychotherapeutic process is associated with better treatment outcome in patients with mood disorders. Psychother Res 2020;30(4): 520–531. doi: 10.1080/10503307.2019.1633484 31256713

[9] Kratzer L, Heinz P. Prozessmonitoring und Feedback in der Psychotraumatologie: Hintergründe und Anwendung. In: Viol K, Schoeller H, Aichhorn W, editors. Selbstorganisation–ein Paradigma für die Humanwissenschaften. Wiesbaden: Springer; 2020. pp. 451–466. 10.1007/978-3-658-29906-4_25

[10] Helmich MA, Wichers M, Olthof M, Strunk G, Aas B, Aichhorn W, et al. Sudden gains in day-to-day change: Revealing nonlinear patterns of individual improvement in depression. J Consult Clin Psychol 2020;88(2): 119–127. doi: 10.1037/ccp0000469 31894994

[11] Kelly MAR, Roberts JE, Ciesla JA. Sudden gains in cognitive behavioral treatment for depression: when do they occur and do they matter? Behav Res Ther 2005;43(6): 703–714. doi: 10.1016/j.brat.2004.06.002 15890164

[12] Olthof M, Hasselman F, Strunk G, van Rooij M, Aas B, Helmich MA, et al. Critical fluctuations as an early-warning signal for sudden gains and losses in patients receiving psychotherapy for mood disorders. Clin Psychol Sci 2019. 10.1177/2167702619865969

[13] Bachler E, Fruehmann A, Bachler H, Aas B, Nickel M, Schiepek G. The effect of childhood adversities and protective factors on the development of child-psychiatric disorders and their treatment. Front Psychol 2018;9(2226). doi: 10.3389/fpsyg.2018.02226 30524336 PMC6262315

[14] Schubert C. Zur Synergetik des systemischen Lupus Erythematodes. In: Viol K, Schoeller H, Aichhorn W, editors. Selbstorganisation–ein Paradigma für die Humanwissenschaften. Wiesbaden: Springer; 2020. pp. 403–421. 10.1007/978-3-658-29906-4_22

[15] Michaelis R. Epilepsy: a window to the investigation of mind-brain interaction. In: Viol K, Schoeller H, Aichhorn W, editors. Selbstorganisation–ein Paradigma für die Humanwissenschaften. Wiesbaden: Springer; 2020. pp. 373–388. 10.1007/978-3-658-29906-4_20

[16] Michaelis R, Schoeller H, Hoeller Y, Kalss G, Kirschner M, Schmid E, et al. Integrating the systematic assessment of psychological states in the epilepsy monitoring unit: Concept and compliance. Epilepsy Behav 2018;88: 5–14. doi: 10.1016/j.yebeh.2018.08.029 30212726

[17] Fartacek C, Plöderl M. The suicidal process: A nonlinear dynamic perspective. In: Viol K, Schoeller H, Aichhorn W, editors. Selbstorganisation–ein Paradigma für die Humanwissenschaften. Wiesbaden: Springer; 2020. pp. 467–476. 10.1007/978-3-658-29906-4_26

[18] Fartacek C, Schiepek G, Kunrath S, Fartacek R, Plöderl M. Real-Time monitoring of nonlinear suicidal dynamics: Methodology and a demonstrative case report. Front Psychol 2016;7(130). 10.3389/fpsyg.2016.00130 PMC4753305 26913016

[19] Kazdin AE. Understanding how and why psychotherapy leads to change. Psychother Res 2009;19(4–5): 418–428. doi: 10.1080/10503300802448899 19034715

[20] Lorenzo-Luaces L, DeRubeis RJ. Miles to go before we sleep: Advancing the understanding of psychotherapy by modeling complex processes. Cog Ther Res 2018;42(2): 212–217. 10.1007/s10608-018-9893-x

[21] Kazdin AE. Mediators and mechanisms of change in psychotherapy research. Ann Rev Clin Psychol 2007;3: 1–27. doi: 10.1146/annurev.clinpsy.3.022806.091432 17716046

[22] Hayes AM, Laurenceau JP, Feldman G, Strauss JL, Cardaciotto LA. Change is not always linear: The study of nonlinear and discontinuous patterns of change in psychotherapy. Clin Psychol Rev 2007;27(6): 715–723. doi: 10.1016/j.cpr.2007.01.008 17316941 PMC3163164

[23] Schiepek G, Gelo O, Viol K, Kratzer L, Orsucci F, Felice G, et al. Complex individual pathways or standard tracks? A data‐based discussion on the trajectories of change in psychotherapy. Couns Psychother Res 2020;20(4): 689–702. 10.1002/capr.12300

[24] Haken H, Schiepek G. Synergetik in der Psychologie: Selbstorganisation verstehen und gestalten [Synergetics in psychotherapy: Understanding and supporting self-organization]. 2nd ed. Göttingen: Hogrefe; 2010.

[25] Schiepek G, Schoeller H, Carl R, Aichhorn W, Lichtwarck-Aschoff A. A nonlinear dynamic systems approach to psychological interventions. In: Kunnen ES, de Ruiter NMP, Jeronimus BF, van der Gaag MAE, editors. Psychosocial development in adolescence: Insights from the dynamic systems approach. New York: Routledge; 2019. pp. 51–68.

[26] Schiepek G, Schoeller H, de Felice G, Steffensen SV, Bloch MS, Fartacek C, et al. Convergent validation of methods for the identification of psychotherapeutic phase transitions in time series of epirical and model systems. Front Psychol 2020;11(1970). 10.3389/fpsyg.2020.01970 PMC7479190 32982834

[27] Haken H. Synergetics. 2nd ed. Berlin: Springer; 1982.

[28] Dakos V, Carpenter SR, Brock WA, Ellison AM, Guttal V, Ives AR, et al. Methods for detecting early warnings of critical transitions in time series illustrated using simulated ecological data. PLoS ONE 2012;7(7):e41010. doi: 10.1371/journal.pone.0041010 22815897 PMC3398887

[29] Livina VN, Kwasniok F, Lohmann G, Kantelhardt JW, Lenton TM. Changing climate states and stability: From Pliocene to present. Climate Dyn 2011;37(11–12): 2437–2453. 10.1007/s00382-010-0980-2

[30] Scheffer M, Bascompte J, Brock WA, Brovkin V, Carpenter SR, Dakos V, et al. Early-warning signals for critical transitions. Nature 2009; 461(7260): 53–59. doi: 10.1038/nature08227 19727193

[31] Killick R, Fearnhead P, Eckley IA. Optimal detection of changepoints with a linear computational cost. J Am Statist Ass 2012;107(500): 1590–1598. 10.1080/01621459.2012.737745

[32] Kowalik Z, Schiepek G, Kumpf K, Roberts L, Elbert T. Psychotherapy as a chaotic process II. The application of nonlinear analysis methods on quasi time series of the client-therapist interaction: A nonstationary approach. Psychother Res 1997;7(3): 197–218. 10.1080/10503309712331331973

[33] Rosenstein MT, Collin JJ, De Luca CJ, Rapp PE. A practical method for calculating largest Lyapunov exponents from small data sets. Physica D 1993;65.

[34] Wolf A, Swift JB, Swinney HL, Vastano JA. Determining Lyapunov exponents from a time series. Physica D 1985;16(3): 285–317. 10.1016/0167-2789(85)90011-9

[35] Skinner JE, Molnar M, Tomberg C. The point correlation dimension: Performance with nonstationary surrogate data and noise. Integr Physiol Behav Sci 1994;29(3): 217–234. doi: 10.1007/BF02691327 7811643

[36] Skinner JE, Molnar M, Vybiral T, Mitra M. Application of chaos theory to biology and medicine. Integr Physiol Behav Sci 1992;27(1): 39–53. doi: 10.1007/BF02691091 1576087

[37] Stockwell RG, Mansinha L, Lowe RP. Localization of the complex spectrum: The S transform. IEEE Trans Signal Proc 1996;44(4): 998–1001. 10.1109/78.492555

[38] Schiepek G, Strunk G. The identification of critical fluctuations and phase transitions in short term and coarse-grained time series—a method for the real-time monitoring of human change processes. Biol Cybern 2010;102(3): 197–207. doi: 10.1007/s00422-009-0362-1 20084517

[39] Coco MI, Mønster D, Leonardi G, Dale R, Wallot S. Unidimensional and multidimensional methods for Recurrence Quantification Analysis with crqa. The R Journal 2021;13(1). doi: 10.32614/RJ-2021-033 34513030 PMC8434812

[40] Webber CL, Marwan N Jr. Recurrence Quantification Analysis: Theory and best practices. Berlin: Springer Series in Understanding Complex Systems. 2015.

[41] Pitsik E, Frolov N, Kraemer KH, Grubov V, Maksimenko V, Kurths J, et al. Motor execution reduces EEG signals complexity: Recurrence quantification analysis study. Chaos 2020;30(2): 023111. doi: 10.1063/1.5136246 32113225

[42] Wallot S, Roepstorff A, Monster D. Multidimensional Recurrence Quantification Analysis (MdRQA) for the analysis of multidimensional time series: A software implementation in MATLAB and its application to group-level data in joint action. Front Psychol 2016; 7(1835). doi: 10.3389/fpsyg.2016.01835 27920748 PMC5118451

[43] Wallot S, Mitkidis P, McGraw JJ, Roepsstorff A. Beyond synchrony: Joint action in a complex production task reveals beneficial effects of decreased interpersonal synchrony. PlosONE 2016;11(12): e0168306. 10.1371/journal.pone.0168306 PMC5172585 27997558

[44] Wallot S. Multidimensional Cross-Recurrence Quantification Analysis (MdCRQA)–A method for quantifying correlation between multivariate time-series. Multivar Behav Res 2018; doi: 10.1080/00273171.2018.1512846 30569740

[45] Lameu EL, Yanchuk S, Macau EEN, Borges FS, Iarosz KC, Caldas IL, et al. Recurrence quantification analysfpsyg.2016is for the identification of burst phase synchronization. Chaos 2018;28: 085701. doi: 10.1063/1.5024324 30180612

[46] Santos MS, Szezech JD Jr, Batista AM, Caldas IL, Viana RL, Lopes SR. Recurrence quantification analysis in chimera states. Phys Lett A. 2015;379: 2188–2192. 10.1016/j.physleta.2015.07.029

[47] Marwan N, Kurths J. Nonlinear analysis of bivariate data with cross recurrence plots. Phys Lett A 2002;302: 299–307. 10.1016/S0375-9601(02)01170-2

[48] Schiepek G, Aas B, Viol K. (2016). The mathematics of psychotherapy–a nonlinear model of change dynamics. Nonlin Dyn Psychol Life Sci 2016;20(3): 369–399. 27262423

[49] Schiepek G, Viol K, Aichhorn W, Huett MT, Sungler K, Pincus D, et al. Psychotherapy is chaotic–(not only) in a computational world. Front Psychol 2017;8(379). 10.3389/fpsyg.2017.00379 PMC5402620 28484401

[50] Schoeller H, Viol K, Aichhorn W, Huett MT, Schiepek G. Personality development in psychotherapy: a synergetic model of state-trait dynamics. Cog Neurodyn 2018;12(5): 441–459. 10.1007/s11571-018-9488-y PMC6139101 30250624

[51] Schoeller H, Viol K, Goditsch H, Aichhorn W, Huett MT, Schiepek G. A nonlinear dynamic systems model of psychotherapy: first steps toward validation and the role of external input. Nonlin Dyn Psychol Life Sci 2019; 23(1): 79–112. 30557137

[52] Derogatis LR, Lipman R, Covi L. SCL-90. Administration, scoring and procedures manual-I for the R (revised) version and other instruments of the psychopathology rating scales series. Baltimore: Johns Hopkins University School of Medicine; 1977.

[53] Kossakowski JJ, Groot PC, Haslbeck JMB, Borsboom D, Wichers M. Data from ‘critical slowing down as a personalized early warning signal for depression’. J Open Psychol Data 2017;5. 10.5334/jopd.29

[54] Wichers M, Groot PC, Wichers M, Borsboom D, Cramer AOJ, Epskamp S, et al. Critical slowing down as a personalized early warning signal for depression. Psychother Psychosom 2016;85(2): 114–116. doi: 10.1159/000441458 26821231

[55] Schiepek G, Stoeger-Schmidinger B, Kronberger H, Aichhorn W, Kratzer L, Heinz P, et al. The Therapy Process Questionnaire—Factor analysis and psychometric properties of a multidimensional self-rating scale for high-frequency monitoring of psychotherapeutic processes. Clin Psychol Psychother 2019;26(5): 586–602. doi: 10.1002/cpp.2384 31153157 PMC6852168

[56] Bandt C, Pompe B. Permutation Entropy: A natural complexity measure for time series. Phys Rev Lett 2002;88(17): 174102. doi: 10.1103/PhysRevLett.88.174102 12005759

[57] Boashash B. Estimating and interpreting the instantaneous frequency of a signal. I. Fundamentals. Proceed IEEE 1992;80(4): 520–538. 10.1109/5.135376

[58] Viol K. Dynamic Complexity Matlab Code. 2019. https://github.com/kviol/synergetik

[59] Marwan N, Romano MC, Thiel M, Kurths J. Recurrence plots for the analysis of complex systems. Phys Rep 2007;438: 237–329.

[60] Takens F. Detecting stange attractors in turbulance. In: Rand D, Young LS, editors. Dynamical systems and turbulence. Berlin: Springer; 1981. pp. 366–381. 10.1007/BFb0091924

[61] Kraemer HK, Donner RV, Heitzig J, Marwan N. Recurrence threshold selection for obtaining robust recurrence characteristics in different embedding dimensions. Chaos 2018;28: 085720. doi: 10.1063/1.5024914 30180619

[62] Chen Y, Yang H. Multiscale recurrence analysis of long-term nonlinear and nonstationary time series. Chaos Solitons Fract 2012;45(7): 978–987. 10.1016/j.chaos.2012.03.013

[63] Yang Hui. Multiscale recurrence quantification analysis of spatial cardiac vectorcardiogram signals. IEEE Trans Biomed Engineering 2011;58(2): 339–347. doi: 10.1109/TBME.2010.2063704 20693104

[64] Cohen L. Time-Frequency Distributions. A review. Proc IEEE 1989;77(7): 941–981. 10.1109/5.30749

[65] Sejdić E, Djurović I, Jiang J. Time-frequency feature representation using energy concentration: An overview of recent advances. Digit Sig Proc 2009;19(1): 153–183.

[66] Sundar A. Time frequency distribution of a signal using S-transform (Stockwell transform). MATLAB Central File Exchange. 2020. https://www.mathworks.com/matlabcentral/fileexchange/51808-time-frequency-distribution-of-a-signal-using-s-transform-stockwell-transform

[67] Heinzel S, Tominschek I, Schiepek G. Dynamic patterns in psychotherapy—discontinuous changes and critical instabilities during the treatment of obsessive compulsive disorder. Nonlin Dyn Psychol Life Sci 2014;18(2): 155–176. 24560009

[68] Schiepek G, Viol K, Aas B, Kastinger A, Kronbichler M, Schoeller H, et al. Pathologically reduced neural flexibility recovers during psychotherapy of OCD patients. NeuroImage:Clin 2021;32(102844). doi: 10.1016/j.nicl.2021.102844 34653839 PMC8527047

[69] an der Heiden U. Chaos in health and disease: Phenomenology and theory. In: Tschacher W, Schiepek G, Brunner E, editors. Self-organization and clinical psychology. Berlin: Springer; 1992. pp. 55–87.

[70] Rensing L, an der Heiden U, Mackey MC. Temporal Disorders in Human Oscillatory Systems. Berlin: Springer; 1987.

