# Supplementary material for: Detecting pattern transitions in psychological time series – A validation study on the Pattern Transition Detection Algorithm (PTDA)
Source: PLoS One. 2022 Mar 11;17(3):e0265335. doi: 10.1371/journal.pone.0265335 (PMC8916631; doi:10.1371/journal.pone.0265335)
Supplement: S1 File — (PDF) [file pone.0265335.s001.pdf]

Supplement to:

Detecting pattern transitions in psychological time series – A validation study on the Pattern Transition Detection Algorithm (PTDA)

Kathrin Viol, Helmut Schöller, Andreas Kaiser, Clemens Fartacek, Wolfgang Aichhorn, Günter Schiepek

1.) Simulation of time series

The following time series were generated by 60 simulation runs of a mathematical model on psychotherapeutic change processes (refer to 2.)). Each run results in five time series (one for each of the five variables that constitute the model). The original data are available as supplement *"SimulationData.csv".* Each column contains a time series with 100 iteration steps each. Of these, five adjacent columns each contain the five variables of a simulation run. In total, the file contains 60 simulations with 5 variables each, i.e. 300 time series (columns).

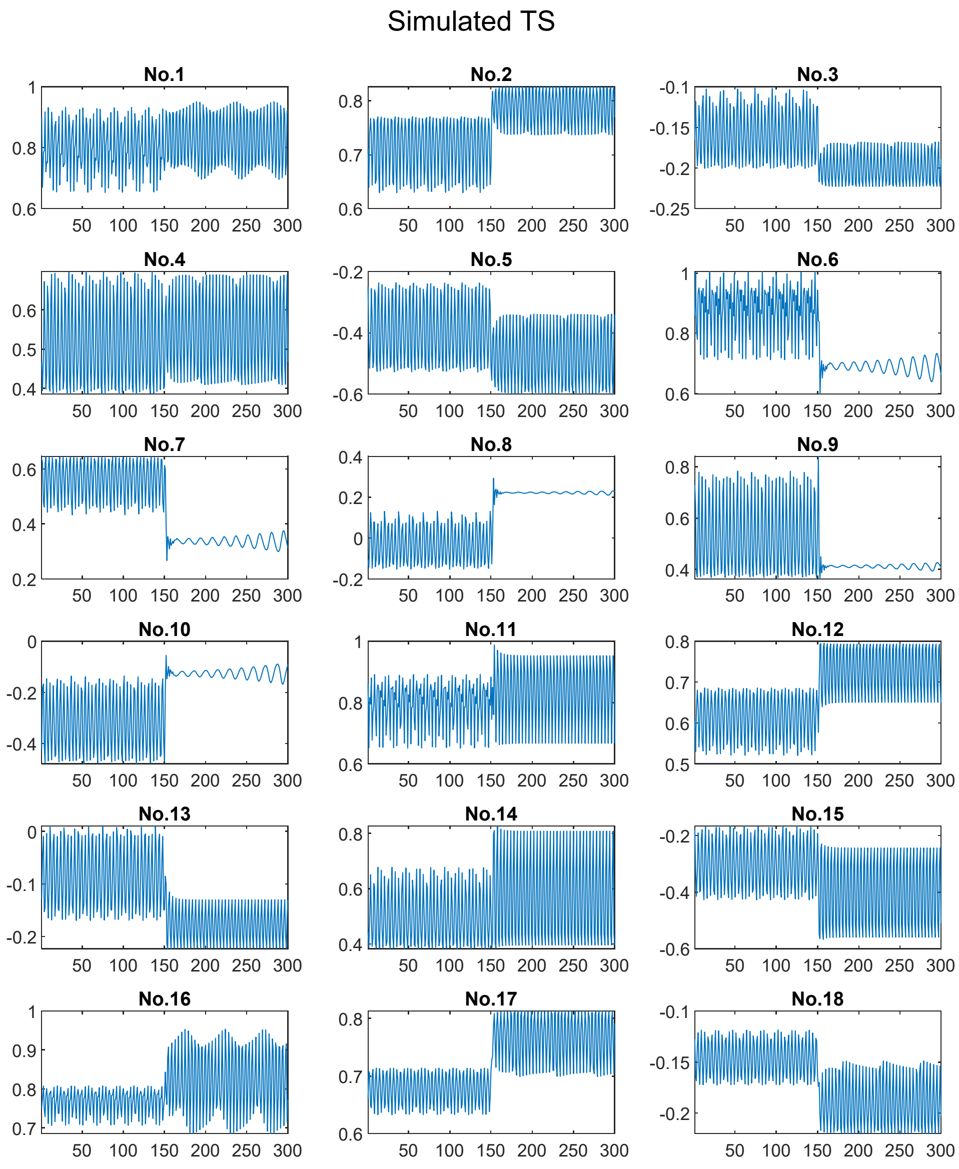

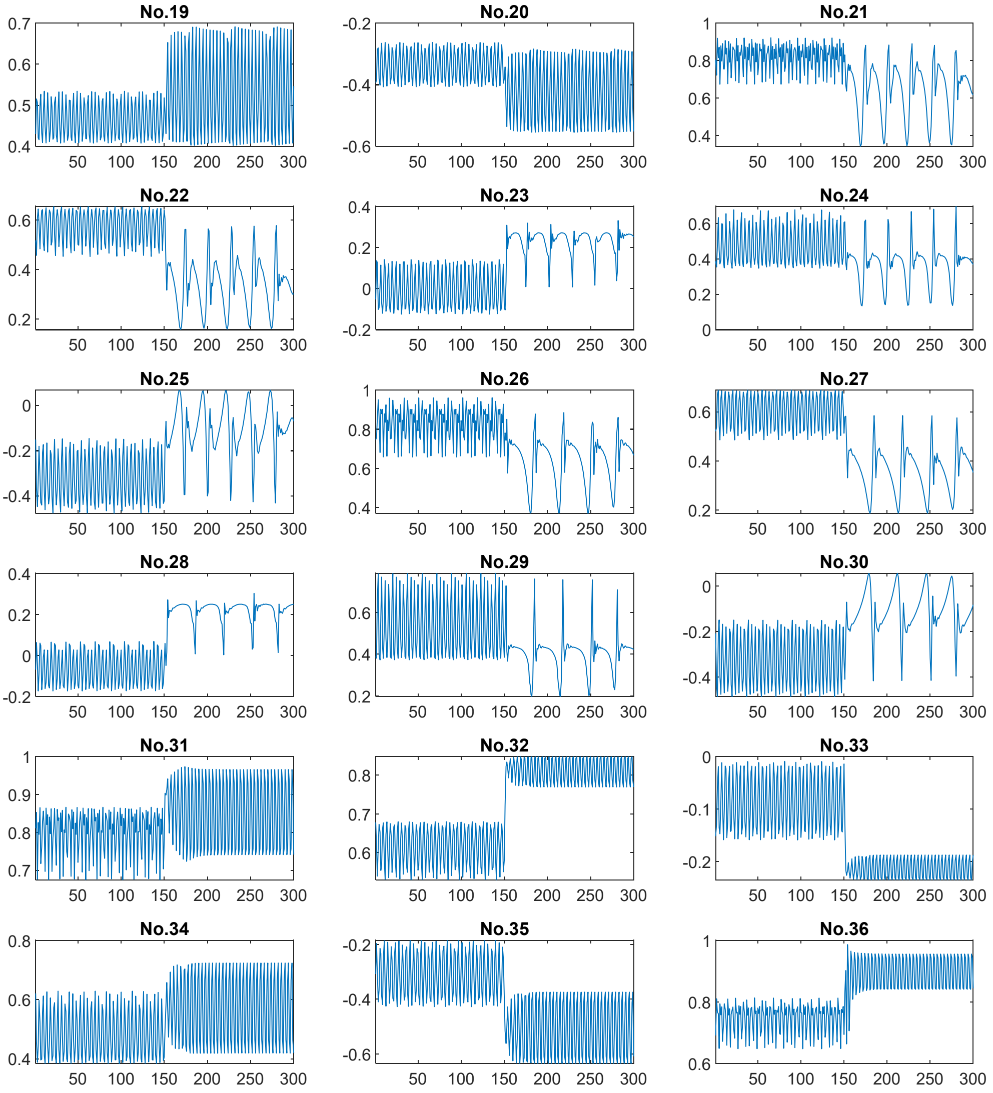
No.19
0.7
0.6
0.5
0.4
50 100 150 200 250 300
No.20
-0.2
-0.4
-0.6
50 100 150 200 250 300
No.21
1
0.8
0.6
0.4
50 100 150 200 250 300
No.22
0.6
0.4
0.2
50 100 150 200 250 300
No.23
0.4
0.2
0
-0.2
50 100 150 200 250 300
No.24
0.6
0.4
0.2
0
50 100 150 200 250 300
No.25
0
-0.2
-0.4
50 100 150 200 250 300
No.26
1
0.8
0.6
0.4
50 100 150 200 250 300
No.27
0.6
0.4
0.2
50 100 150 200 250 300
No.28
0.4
0.2
0
-0.2
50 100 150 200 250 300
No.29
0.6
0.4
0.2
50 100 150 200 250 300
No.30
0
-0.2
-0.4
50 100 150 200 250 300
No.31
1
0.9
0.8
0.7
50 100 150 200 250 300
No.32
0.8
0.7
0.6
50 100 150 200 250 300
No.33
0
-0.1
-0.2
50 100 150 200 250 300
No.34
0.8
0.6
0.4
50 100 150 200 250 300
No.35
-0.2
-0.4
-0.6
50 100 150 200 250 300
No.36
1
0.8
0.6
50 100 150 200 250 300

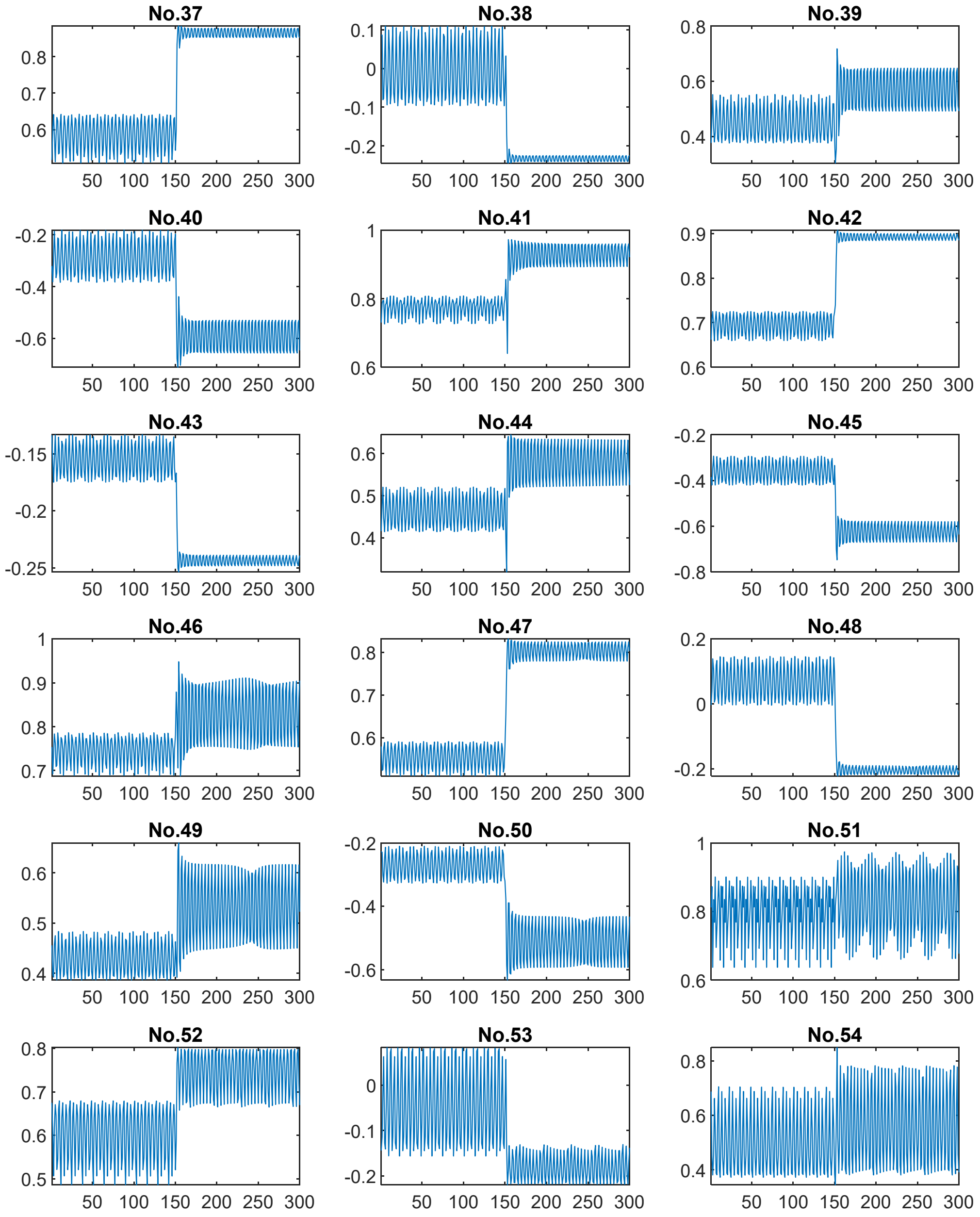
No.37
No.38
No.39
No.40
No.41
No.42
No.43
No.44
No.45
No.46
No.47
No.48
No.49
No.50
No.51
No.52
No.53
No.54

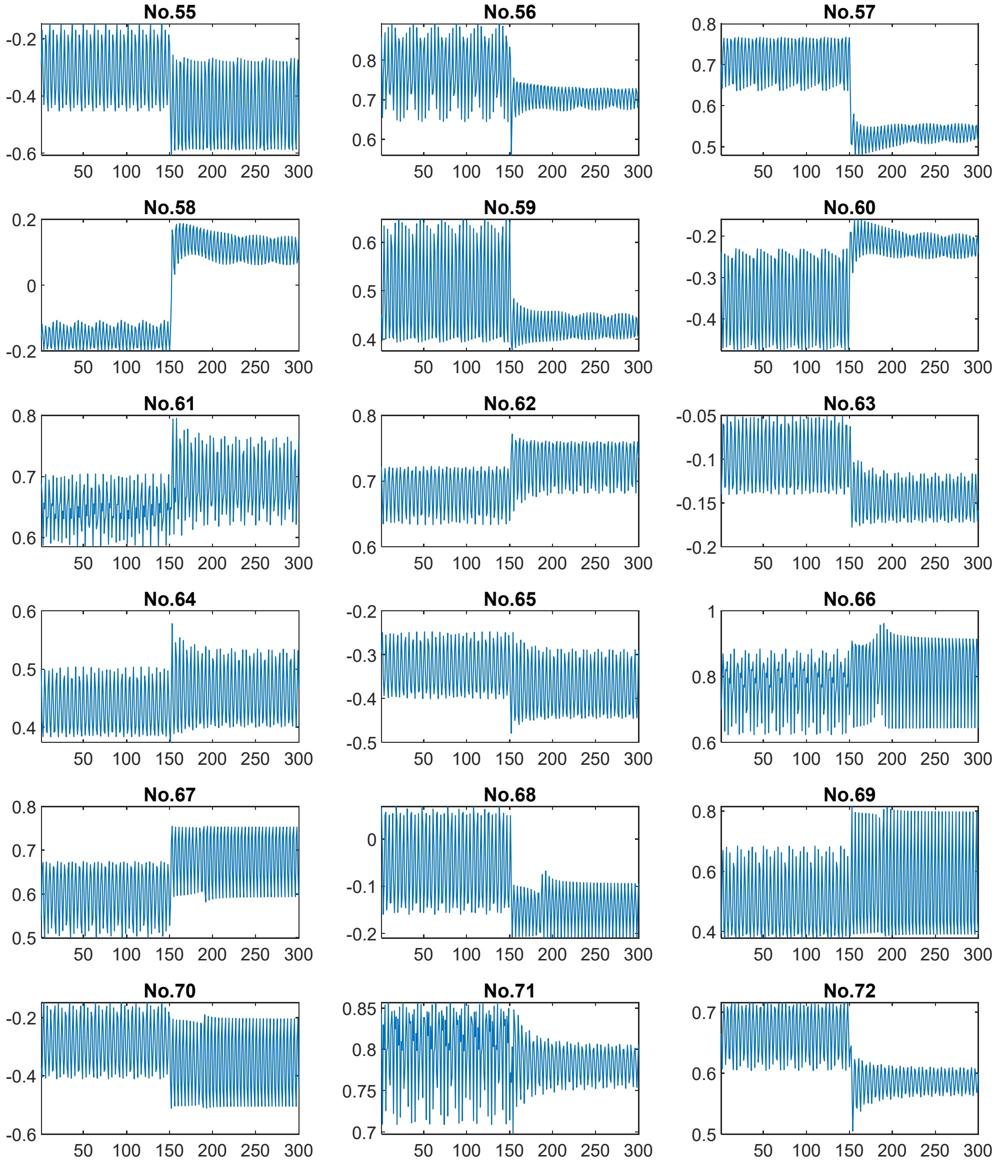
No.55
-0.2 -0.4 -0.6
No.56
0.8 0.7 0.6
No.57
0.8 0.7 0.6 0.5
No.58
0.2 0 -0.2
No.59
0.6 0.5 0.4
No.60
-0.2 -0.3 -0.4
No.61
0.8 0.7 0.6
No.62
0.8 0.7 0.6
No.63
-0.05 -0.1 -0.15 -0.2
No.64
0.6 0.5 0.4
No.65
-0.2 -0.3 -0.4 -0.5
No.66
1 0.8 0.6
No.67
0.8 0.7 0.6 0.5
No.68
0 -0.1 -0.2
No.69
0.8 0.6 0.4
No.70
-0.2 -0.4 -0.6
No.71
0.85 0.8 0.75 0.7
No.72
0.7 0.6 0.5
50 100 150 200 250 300

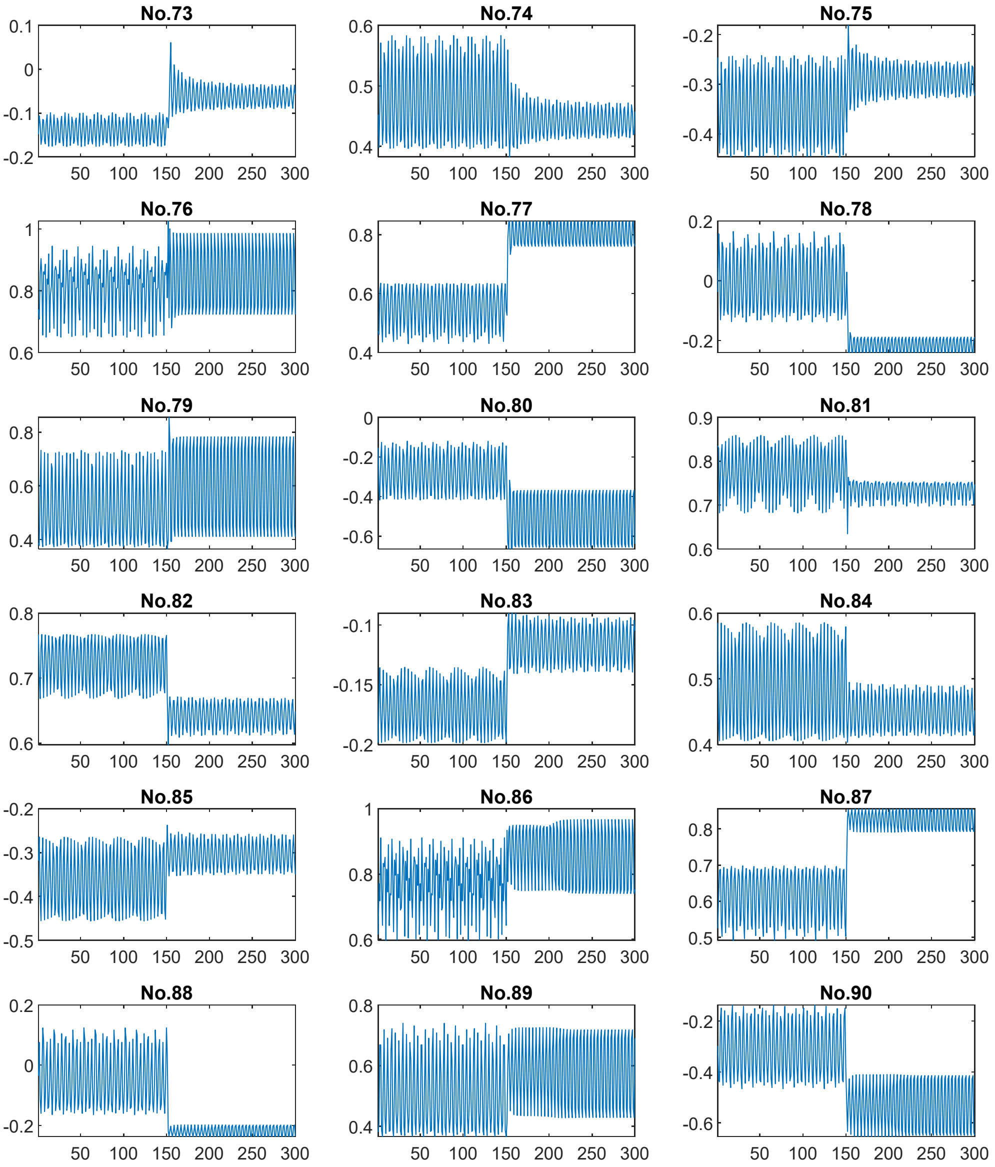
No.73
No.74
No.75
No.76
No.77
No.78
No.79
No.80
No.81
No.82
No.83
No.84
No.85
No.86
No.87
No.88
No.89
No.90

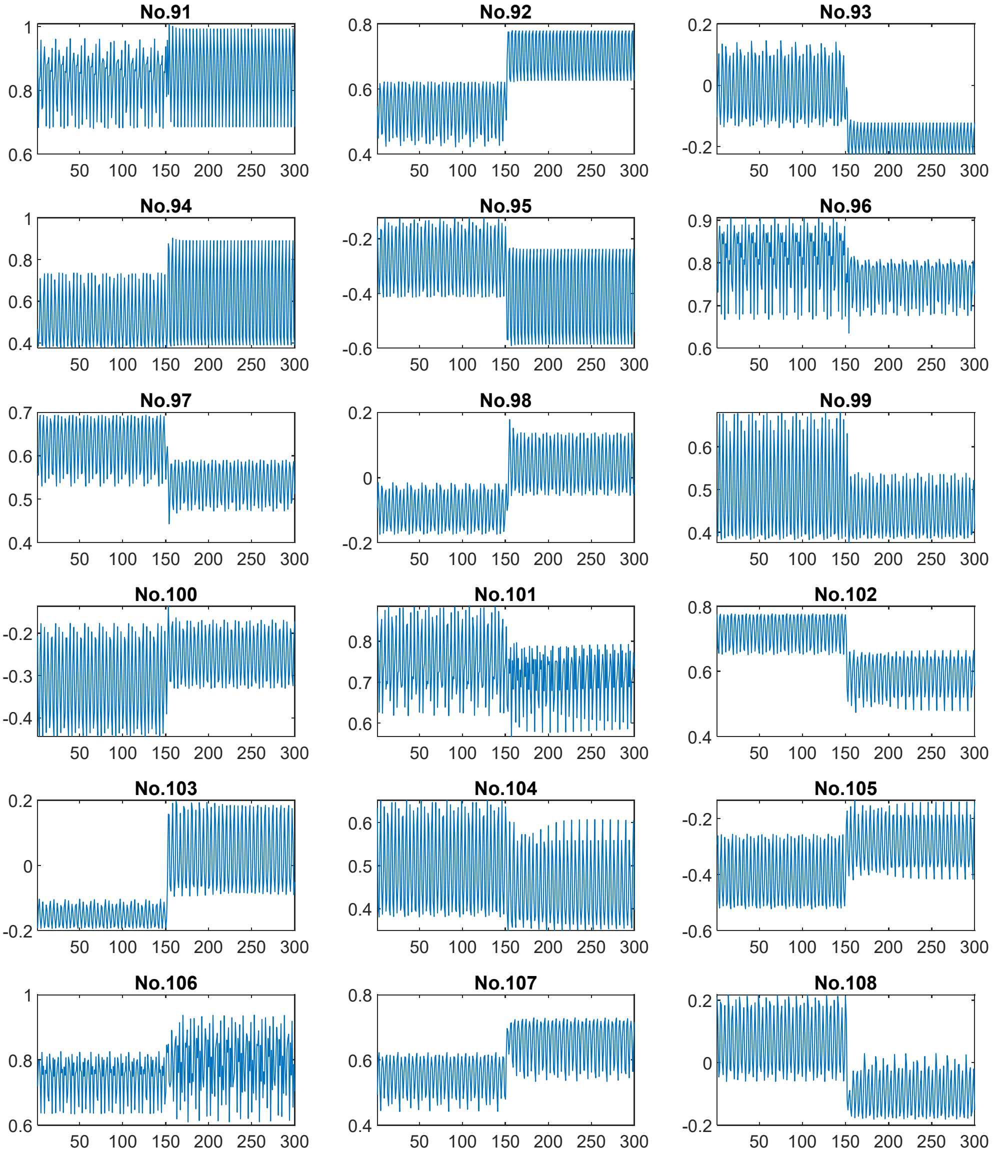
No.91
No.92
No.93
No.94
No.95
No.96
No.97
No.98
No.99
No.100
No.101
No.102
No.103
No.104
No.105
No.106
No.107
No.108

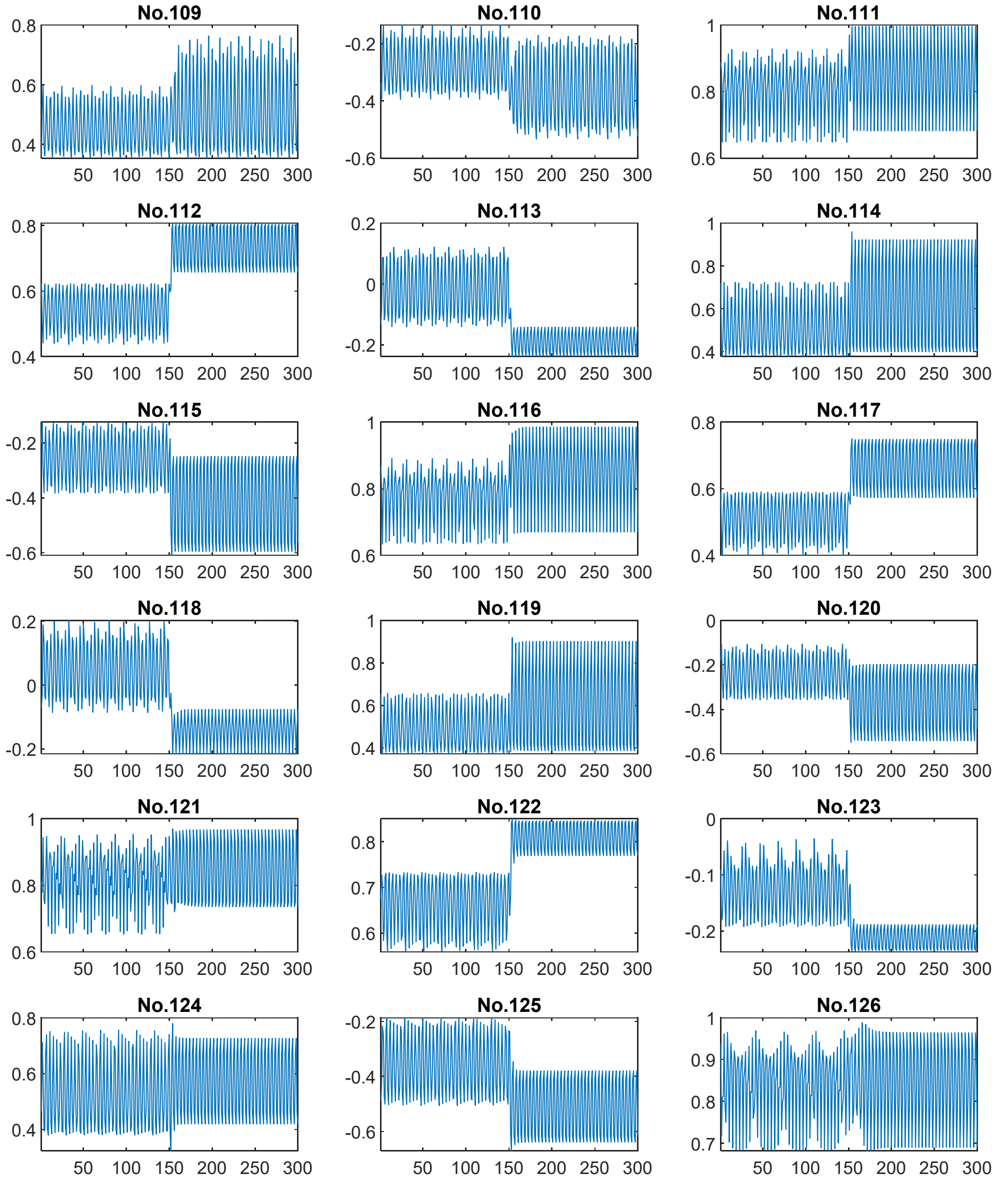
No.109
No.110
No.111
No.112
No.113
No.114
No.115
No.116
No.117
No.118
No.119
No.120
No.121
No.122
No.123
No.124
No.125
No.126

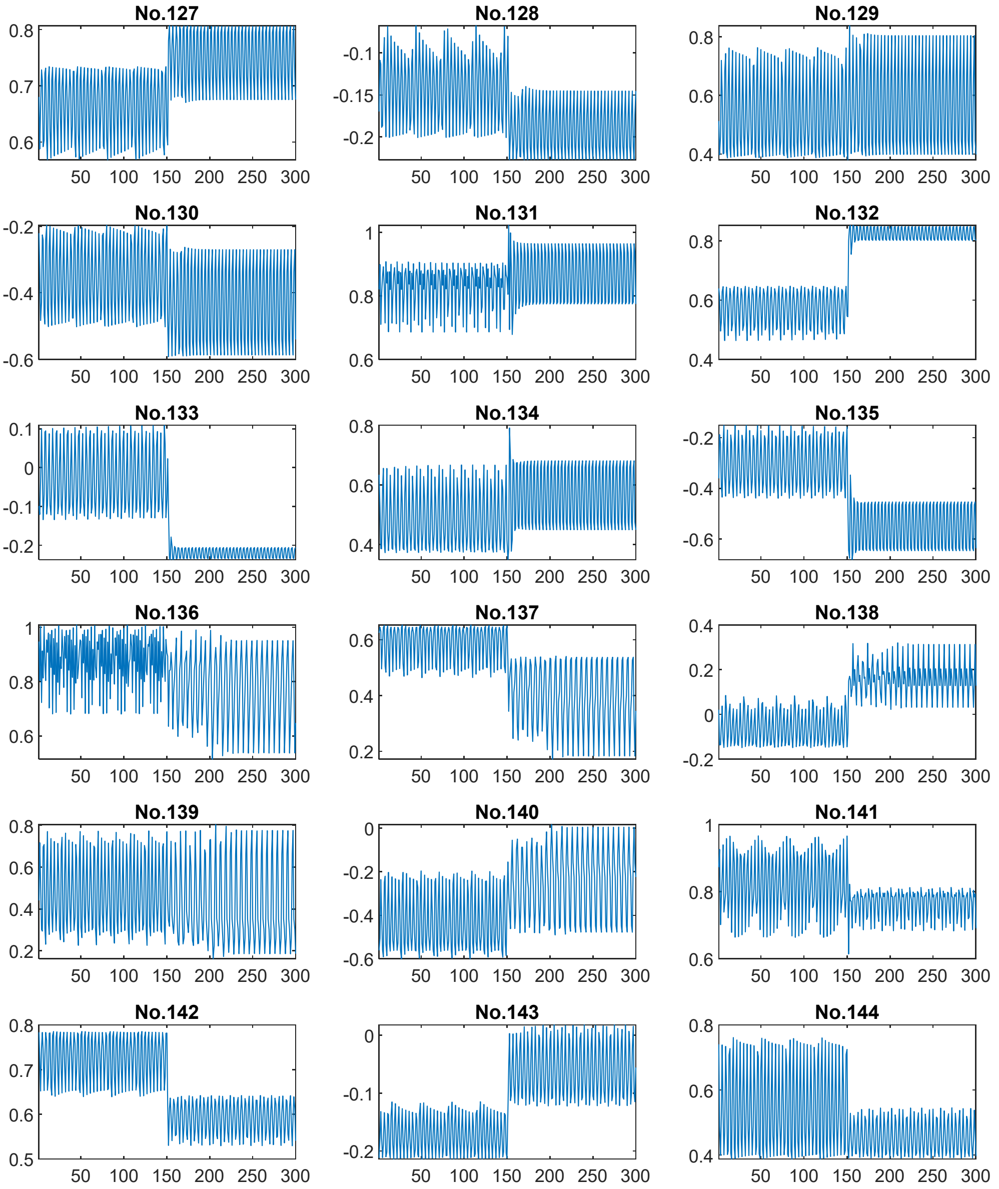
No.127
0.8 0.7 0.6
50 100 150 200 250 300
No.128
-0.1 -0.15 -0.2
50 100 150 200 250 300
No.129
0.8 0.6 0.4
50 100 150 200 250 300
No.130
-0.2 -0.4 -0.6
50 100 150 200 250 300
No.131
1 0.8 0.6
50 100 150 200 250 300
No.132
0.8 0.6 0.4
50 100 150 200 250 300
No.133
0.1 0 -0.1 -0.2
50 100 150 200 250 300
No.134
0.8 0.6 0.4
50 100 150 200 250 300
No.135
-0.2 -0.4 -0.6
50 100 150 200 250 300
No.136
1 0.8 0.6
50 100 150 200 250 300
No.137
0.6 0.4 0.2
50 100 150 200 250 300
No.138
0.4 0.2 0 -0.2
50 100 150 200 250 300
No.139
0.8 0.6 0.4 0.2
50 100 150 200 250 300
No.140
0 -0.2 -0.4 -0.6
50 100 150 200 250 300
No.141
1 0.8 0.6
50 100 150 200 250 300
No.142
0.8 0.7 0.6 0.5
50 100 150 200 250 300
No.143
0 -0.1 -0.2
50 100 150 200 250 300
No.144
0.8 0.6 0.4
50 100 150 200 250 300

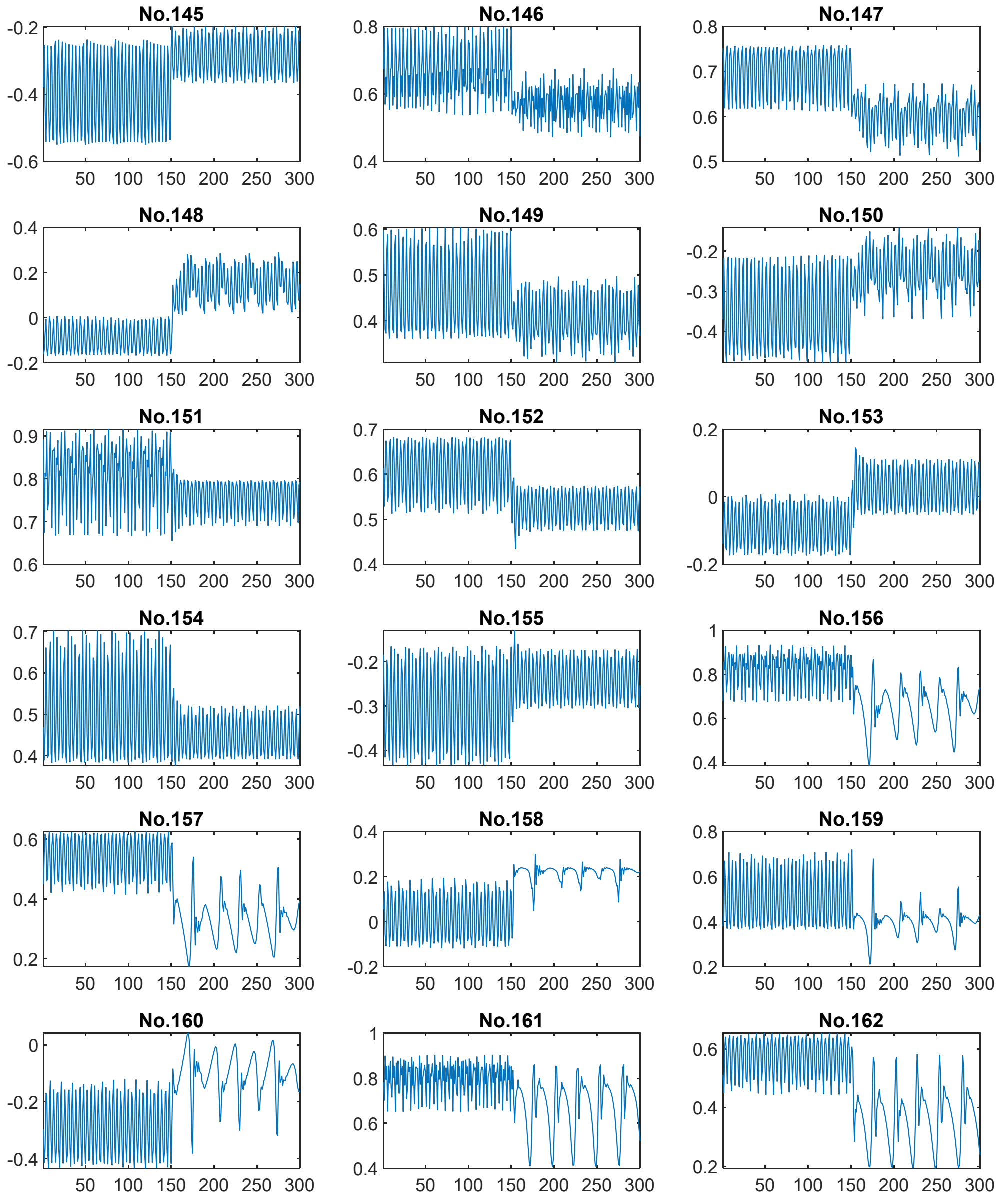
No.145
No.146
No.147
No.148
No.149
No.150
No.151
No.152
No.153
No.154
No.155
No.156
No.157
No.158
No.159
No.160
No.161
No.162
50 100 150 200 250 300

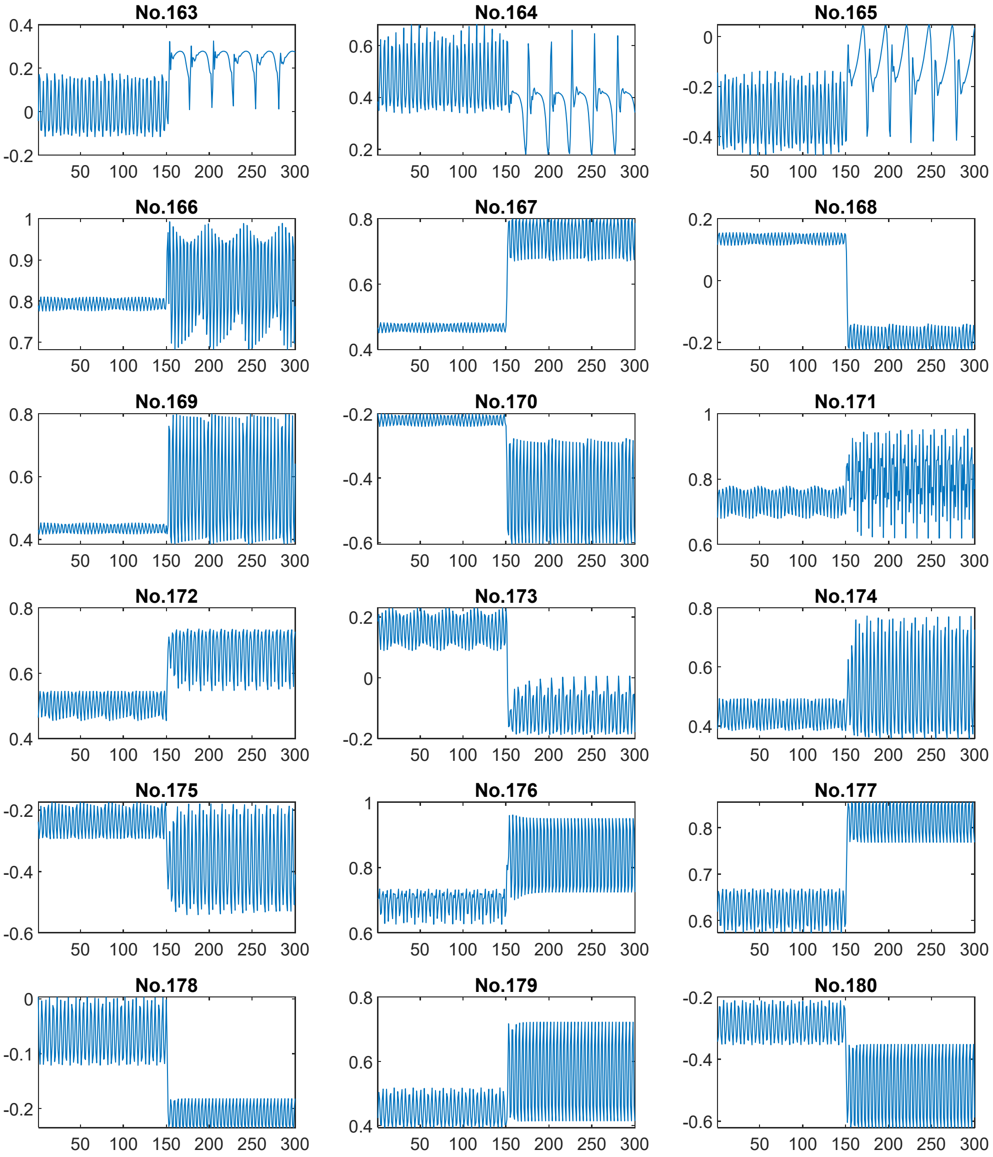
No.163
No.164
No.165
No.166
No.167
No.168
No.169
No.170
No.171
No.172
No.173
No.174
No.175
No.176
No.177
No.178
No.179
No.180

2.) Simulation model

The nine coupled non-linear difference equations of the respective simulation model. Equations 1 to 5 represent five variables, equations 6 to 9 calculate the time course of the parameter values, if the automatic parameter calculation is applied. In this case, the initial values for a(0), c(0), r(0) and m(0) must be specified.

For further details please refer to Schöller H, Viol K, Aichhorn W, Hütt MT, Schiepek G. Personality development in psychotherapy: a synergetic model of state-trait dynamics. Cogn Neurodyn. 2018 Oct;12(5):441-459. doi: 10.1007/s11571-018-9488-y. Epub 2018 Jun 4. PMID: 30250624; PMCID: PMC6139101.

1. $E(E,I,P,S,c,r,m) = \frac{1}{1+e^{-10E}} - c + \frac{1}{1+e^{-20I\cdot(1-\frac{c+r}{2})+5}} + \frac{\frac{-1}{1+e^{\left(2+3\cdot\left(1-\frac{c+m}{2}\right)\right)\cdot P}}+0.5+0.5\cdot\left(1-\frac{c+m}{2}\right)}{1+e^{25\cdot\left(1-\frac{c+m}{2}\right)\cdot\left(P-0.2-0.75\cdot\left(1-\frac{c+m}{2}\right)\right)}} + \frac{1.25}{1+e^{5S-0.5}} - 0.5 - 0.5\,m$
2. $\mathrm{I}(\mathrm{E},\mathrm{M},\mathrm{S},\mathrm{a},\mathrm{c}) = \frac{1}{1+e^{-20E\cdot\left(\frac{a+c}{2}\right)+5}} + \frac{1}{1+e^{-20M\cdot(\frac{a+c}{2})+5}} + \frac{1}{1+e^{-20\cdot|S|\cdot c+5}}$
3. $\mathrm{M}(\mathrm{P},\mathrm{S},\mathrm{r},\mathrm{m}) = \frac{1.261}{1+e^{(P-0.05-0.85m)\cdot(10.1+19.9m)}} \cdot \frac{1}{1+e^{-(P-0.43+0.03m)\cdot(7-3m)}} - \frac{1}{1+e^{5S}} + \frac{r+m}{2}$
4. $P(E,S,c,r) = \frac{1}{1+e^{-10E}} - c + \frac{1.2}{1+e^{5S-0.5}} - 0.2 - 0.8\,r$
5. $S(E,I,M,P,S,a,c,m,r) = \frac{1.3}{1+e^{5E-0.5}} - 0.65 + 0.35\cdot(c+m-1) + \frac{1}{1+e^{-20I\cdot\frac{(a+m+r)}{3}+5}} + + \frac{1}{1+e^{-20M\cdot\frac{(a+m+r)}{3}+5}} - - \frac{1}{1+e^{20M\cdot\left(1-\frac{a+m+r}{3}\right)+5}} + \frac{1.25}{1+e^{5P-0.5}} - 0.5 - 0.5\cdot\left(1-\frac{c+m}{2}\right) + \frac{1}{1+e^{-10S}} + + \frac{m+r}{2} - 1$
6. $a_t = a_{t-1} + s_a \cdot w_a \cdot a_{t-1} \cdot \frac{1}{2}\left(f_{S,t,n} - f_{E,t,n}\right)$
7. $c_t = c_{t-1} + s_c \cdot w_c \cdot c_{t-1} \cdot \frac{1}{3}\left(f_{I,t,n} + f_{S,t,n} + r_{t-1}\right)$
8. $r_t = r_{t-1} + s_r \cdot w_r \cdot r_{t-1} \cdot \frac{1}{2}\left(f_{S,t,n} + c_{t-1}\right))$
9. $m_t = m_{t-1} + s_m \cdot w_m \cdot m_{t-1} \cdot \frac{1}{4}\left(-f_{E,t,n} - f_{P,t,n} + f_{M,t,n} + f_{S,t,n}\right)$

3.) Empirical data

The empirical data for Fig. 6 are available as data file "*EmpiricalData.csv*". The column headings contain the references A, B, C and D to the parts of Figure 6.

4.) Illustrative Example for the Calculation of Change Point Analysis (CPA)

This is an illustrative example for the calculation of Change Point Analysis (CPA). Consider the time series {2,2,2,4,4,4,4,4,4,4}, where the mean changes from 2 to 4 between t=3 and t=4. The change point analysis algorithm first splits the time series into two segments, x1 from t=1 to t=2, and x2 from t=3 to t=10. For both segments, the cost function C is calculated: the first part includes N = 2 time points, the second part N = 8 time points with var(x1) = 0 and var(x2) = 0.5, hence C(x1)=2·0=0 and C(x2)=8·0.5=4. The sum of C(x1) and C(x2), 4, is then compared to the cost function of the whole time series, C(x)=10·0.933=9.33. Since C(x1) + C(x2) are not less than C(x), the algorithm concludes there is no change point when segmenting the time series after t=2. It then proceeds by splitting the time series between t=3 and t=4 and repeats the tests for these segments. Now, both the variance of x1 and x2 are zero, hence C(x1)+C(x2)<C(x); a change point is detected correctly between t=3 and t=4. The nine coupled non-linear difference equations of the respective simulation model. Equations 1 to 5 represent five variables, equations 6 to 9 calculate the time course of the parameter values, if the automatic parameter calculation is applied. In this case, the initial values for a(0), c(0), r(0) and m(0) must be specified.
For further details please refer to Schöller H, Viol K, Aichhorn W, Hütt MT, Schiepek G. Personality development in psychotherapy: a synergetic model of state-trait dynamics. Cogn Neurodyn. 2018 Oct;12(5):441-459. doi: 10.1007/s11571-018-9488-y. Epub 2018 Jun 4. PMID: 30250624; PMCID: PMC6139101.

5.) The Dynamic Complexity algorithm

Fluctuation

The fluctuation algorithm is applied to segments of discrete time-series. These segments are defined by the width of a moving window that can be fixed arbitrarily. The window runs over the whole time series and results in a continuous fluctuation intensity measure *F*. All measurement points within the window are subdivided into periods with cut-off points defined by changes in slope (points of return *k*). Trends can be: “increasing”, “decreasing”, or “no change” (Fig. S1). The difference between the values x_n at the points of return *k* is taken irrespective of the sign – in absolute terms: $y_i = \left| x_{n_{k+1}} - x_{n_k} \right|$ – and is divided by the duration of the period (i.e., the number of data points within the period from one point of return *k* to the next one *k*+1). By this, the change rate is related to its duration, and *F* is sensitive to the frequency as well as to the amplitude of the fluctuation. Theses fractions are summed up within the window. In order to normalize the fluctuation intensity, the result is related to the greatest possible fluctuation which is given by the maximum amount of change within a minimum duration. This is the sum of the differences between the lowest and the highest value of the available range between one and the next measurement point. The formula results in a normalized fluctuation intensity $0 \le F \le 1$:

$$F = \frac{\sum_{i=1}^{I} \frac{y_i}{(n_{k+1} - n_k)}}{s(m-1)}$$

with

$$y_i = \left| x_{n_{k+1}} - x_{n_k} \right|$$

x_n *n*th value of the time series

k points of return (number of changes in slope of the data sequence)

i periods between points of return

I total number of such periods within the window

m number of measurement points within a moving window

m–1 number of intervals between all measurement points of a window

$s = x_{max} - x_{min}$ with x_{min} (smallest value of the scale), x_{max} (largest value of the scale).

One can immediately derive that $\sum_{i=1}^{I} \frac{y_i}{n_{k+1} - n_k} \leq s(m-1)$, so $0 \leq F \leq 1$ (see figure 35).

Note that *s*(*m*-1) is the window area expressed in units of *t* and *x* and that *s* is not the empirical data range but the theoretical range of the scale.

Supplement Figure S1

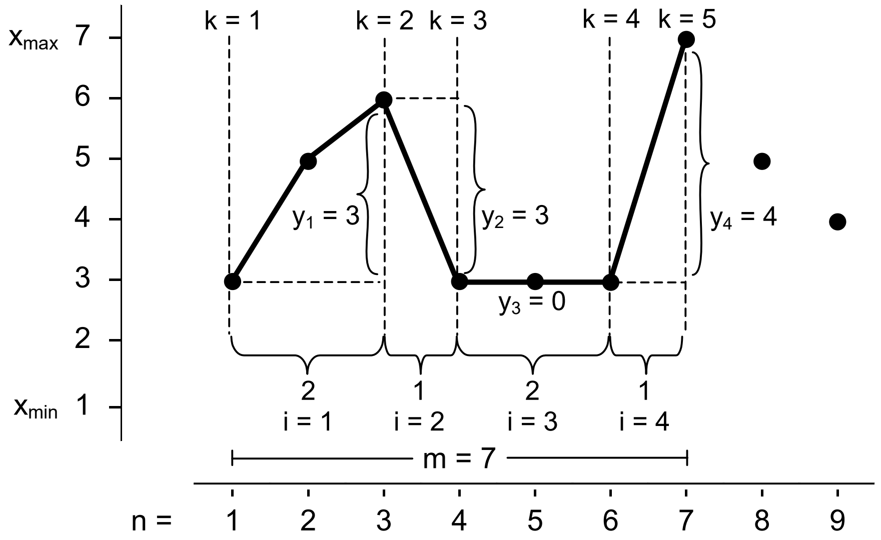

Figure S1: In this example, the *F*-value is calculated as follows: The first contributor is between *k*=1 and *k*=2 a difference of 3 (since $x_1 = 3$, $x_3 = 6$), divides by 2 (2 is the number of intervals between *k*=1 and *k*=2. The next contributor is between *k*=2 and *k*=3 a difference of 3, divided by only one interval. Next is a difference of 0 divided by 2 intervals (remains 0), and the last difference between *k*=4 and *k*=5 is 4, divided by 1. So we sum up 3/2 + 3/1 + 0/2 + 4/1 = 8.5. This sum is divided by the maximum of possible fluctuation, which is in this case (with the greatest number of points of return *k* = 1 to *k* = 7, and $s = (x_{max} - x_{min}) = 7 - 1 = 6$): 6/1 + 6/1 + 6/1+ 6/1 + 6/1 + 6/1 = 36. F = 8.5/36 = .23611.

Distribution

The degree of distribution *D* represents another aspect of critical instabilities. Whereas *F* is at its maximum when the dynamics jump between the minimum and maximum values with greatest and equal frequency, instabilities are often characterized by irregularities, resulting in quite different system states. In the extreme case, the values should be irregularly and chaotically distributed across the range of the measurement scale. As a result, the degree of distribution measures the deviance of the values from an ideal equal distribution of the values across the range or measurement scale. As for the calculation of *F*, we used a moving window running through the whole process and by doing this we consider the values over the full course of the process. For the distribution measure the order of values within the moving window is irrelevant, and in a first step values are sorted in ascending order. Let x_i be the values of a variable *x* at the sorting position *i* within the moving window *X* which is given by:

$X = \{x_1, x_2, x_3, \ldots x_m\}$ with $x_1 \leq x_2 \leq x_3 \leq \ldots x_m$

In the following calculation this sorted window is compared with an artificial data set of equally distributed values. This artificial data set consists of the same number *m* of values arranged in ascending order in equally spaced intervals between the theoretical scale minimum and maximum. The interval *I* is given by $I = s / (m-1)$, $s = x_{max} - x_{min}$ and the artificial data set *Y* is given by:

$Y = \{y_1 = I*1, y_2 = I*2, y_3 = I*3, \ldots y_m = I*m\}$

If the data set in *X* is equally distributed within the data range, then differences between values at different positions in *X* must be equal to the differences in *Y* at the same positions. To give an example, if *X* is perfectly equally distributed within the data range, then $\delta_{Y.2\text{-}1} = y_2 - y_1 = \delta_{X.2\text{-}1} = x_2 - x_1$. Generally the aberration Δ_{ba} of *X* from the ideal given in *Y* can be calculated for the positions *a* and *b* as follows:

$\Delta_{ba} = \delta_{Y.b\text{-}a} - \delta_{X.b\text{-}a}$ with $\delta_{Y.b\text{-}a} = y_b - y_a$ and $\delta_{X.b\text{-}a} = x_b - x_a$

In total the aberration Δ* is given by the following permutation of a and b.

$$\Delta^* = \sum_{c=1}^{m-1} \sum_{d=c+1}^{m} \sum_{a=c}^{d-1} \sum_{b=a+1}^{d} \Delta_{ba} \Theta(\Delta_{ba})$$

The two outer sums are permutations of all combinations of *c* and *d* within the window. The inner sums of *a* and *b* are representing all combinations of positions within the interval given by *c* and *d*.

$\Theta(\Delta_{ba})$ is the Heaviside step function resulting in 1 if Δ_{ba} is a positive number; otherwise the function results in 0. Therefore, only positive aberrations are considered, because negative aberrations have the consequence of resulting in positive ones in other positions.

Hence, the distribution measurement *D* is given by:

$$D = 1 - \sum_{c=1}^{m-1} \sum_{d=c+1}^{m} \sum_{a=c}^{d-1} \sum_{b=a+1}^{d} \frac{\Delta_{ba} \Theta(\Delta_{ba})}{\delta_{Y.ba}}$$

One can see that *D* is normalized so that $0 \leq D \leq 1$, and a high value of *D* are the result of equally distributed measures of *x* within the moving window.
